# The prevalence and risk factors of anxiety in multiple sclerosis: A systematic review and meta-analysis

**DOI:** 10.3389/fnins.2023.1120541

**Published:** 2023-04-17

**Authors:** Xiaoyun Zhang, Ying Song, Zhiqiang Wei, Xiao Chen, Xiaojia Zhuang, Li Yi

**Affiliations:** ^1^Neurology Department, Peking University Shenzhen Hospital, Shenzhen, China; ^2^Rehabilitation Department, Shenzhen Longhua District Central Hospital, Shenzhen, China

**Keywords:** multiple sclerosis, anxiety, prevalence, risk factor, meta-analysis

## Abstract

**Background:**

Patients with multiple sclerosis (MS) suffer from repetitive neurological deterioration, while anxiety may play a significant role in the disease’s progression.

**Objective:**

To explore the prevalence of anxiety in MS and to investigate the risk factors related to anxiety in MS patients.

**Methods:**

An analysis of four databases, PubMed, Web of Science, EMBASE, and Cochrane Library, has been conducted to determine the prevalence or risk factors for anxiety in MS published before May 2021.

**Results:**

In total, 32 studies were found to be eligible. Anxiety prevalence was estimated to be 36% based on the pooled estimates [the 95% confidence interval (CI) = [0.30–0.42], *I*^2^ = 98.4%]. Significant risk factors for developing of anxiety were as follows: age at survey [the weighted mean difference (WMD) = 0.96, 95% CI = [0.86–1.06], *I*^2^ = 43.8%], female [the odd ratio (OR) = 1.78, 95% CI = [1.38–2.30], *I*^2^ = 0%], living together (OR 2.83, 95% CI = [1.74–4.59], *I*^2^ = 0%), past psychiatric history (OR 2.42, 95% CI = [1.56–3.75], *I*^2^ = 0%), depression (OR 7.89, 95% CI = [3.71–16.81], *I*^2^ = 0%), not taking MS medication (OR 2.33, 95% CI = [1.29–4.21], *I*^2^ = 77.8%), relapsing-remitting MS (RRMS) (OR 1.50, 95% CI = [0.94–2.37], *I*^2^ = 53.5%), and baseline Expanded Disability Status Scale (EDSS) (OR 0.84, 95% CI = [0.48–1.21], *I*^2^ = 62.2%).

**Conclusion:**

An estimated 36% of people with MS suffer from anxiety. And anxiety rates in MS patients are significantly associated with age, gender, living together, prior psychiatric history, depression, drug compliance, RRMS, and baseline EDSS.

**Systematic review registration:**

https://www.crd.york.ac.uk/PROSPERO/display_record.php?RecordID=287069, identifier CRD42021287069.

## Background

The prevalence of MS varies between high-income countries and low-income countries, with a global prevalence of 30 cases per 100,000 population (GBD 2016 Multiple Sclerosis Collaborators, 2019) and world-wide prevalence of 60–120 per 100,000 in working-age adults (Levi et al., 2021), imposing a deterioration of the economy and health care (Browne et al., 2014). There are various neurological deficits associated with multiple sclerosis (MS), such as motor, sensory, cognitive and neuropsychiatric impairments.

According to the clinical course, MS can be classified into clinically isolated syndrome (CIS), relapsing-remitting multiple sclerosis (RRMS), secondary progressive MS (SPMS), primary progressive MS (PPMS), and progressive relapsing MS (PRMS) (Kamm et al., 2014). The most common form is RRMS, affecting around 80% of MS patients (Compston and Coles, 2008). RRMS patients can recover to function for an unpredictable period of time before further attacks lead to progressive deterioration. It is important to note, however, that being disease-free does not necessarily mean that patients with RRMS are symptom-free, because they can still suffer from debilitating condition, such as anxiety and depression. Comorbid anxiety and depression are highly prevalent in chronically ill patients (Scott et al., 2007). Given the unpredictable and fluctuating nature of RRMS, it is not surprising that patients may be anxious about when there will be another episode. In addition, anxiety had a greater impact on disease symptoms as compared to depression. Those with anxiety were much more likely to report fatigue, pain, and sleep problems even with no association with depression (Hanna and Strober, 2020).

Anxiety is a mental state characterized by worry or fear in the face of a stressful event, and is not uncommon for patients with MS. Reports on MS and anxiety started around the 1980s with several studies suggesting that anxiety can reach a lifetime prevalence of up to 50% for MS patients (Olivera et al., 1988). Many factors can lead to anxiety, and ultimately cause poor outcomes in social support and disease duration (Hanna and Strober, 2020). Besides, anxiety, depression and pain are significantly associated with the severity of wheelchair dependence in patients with MS (Janssens et al., 2004). And the negative effect of anxiety on the quality of life (QOL) is the most frequently reported outcome in MS patients (van Tilburg et al., 2021).

Early identification and treatment of anxiety may improve working productivity and extend employment for MS patients (Glanz et al., 2012). However, despite early studies have focused on the prevalence of anxiety and depression in MS, few reviews summarized the risk factors relevant to anxiety in MS patients.

This meta-analysis aims to provide an overview of anxiety prevalence in MS patients and to investigate risk factors that are associated with anxiety development. And to our knowledge, our meta-analysis would be the first to discuss the risk factors for anxiety in MS patients.

## Methods

The present systematic review and meta-analysis was conducted in accordance with the Preferred Reporting Items for Systematic Reviews and Meta-Analysis (PRISMA) guidelines (Moher et al., 2009) and has been registered in the PROSPERO database (No. CRD42021287069).

### Search strategy

In order to identify studies discussing anxiety among MS patients, articles published before 16th May 2021 were searched in international databases including PubMed, Web of Science, EMBASE, and Cochrane Library. Search items and synonyms were based on the PECO acronym (population: patients with MS, exposure: risk factors and prevalence, outcome: anxiety) and the Boolean operators “AND” and “OR.”

As our main focus was set on the MS population, studies with a healthy control group were excluded. Detailed search terms are provided in Supplementary Appendix 1. References of eligible studies were also assessed for additional citations.

### Eligibility criteria

The following criteria had to be met in order for a study to be included:

1. Inclusion criteria
  Subjects diagnosed with MS by neurologists fulfilling Poser Criteria (Poser et al., 1983), or original or revised McDonald diagnostic criteria for MS (criteria of 2001 or revised McDonald criteria of 2005/2010/2017), or a clinically definite MS based on retrospective medical records;
  1.1 There should be data available to extract to determine the prevalence or risk factors of anxiety in MS, with the diagnosis criteria for anxiety outlined in Table 1;

**TABLE 1:** Characteristics of included studies.

References	Article type	Nation	Total (*N*)	RR patients (*N*)	Age (*M* ± SD)	M/F	Measurement tool
Askari et al., 2014	Cross-sectional study	Iran	180	142	32.4 ± 8.7	84/236	BAI
Chertcoff et al., 2020	Cross-sectional study	Argentina	83	61	46 ± 13.9	30/53	HADS-A ≥ 8
Garfield and Lincoln, 2012	Cross-sectional study	UK	159	83	50 ± 10.15	47/112	HADS-A ≥ 8
Gascoyne et al., 2019	Cross-sectional study	Australia	1500	858	56 ± 11.2	308/1192	HADS-A ≥ 8
Gay et al., 2017	Cross-sectional study	France	189	107	47.2 ± 12.5	68/121	HADS-A ≥ 8
Gay et al., 2010	Cross-sectional study	France	115	NA	47.22 ± 10.93	36/79	HADS-A ≥ 8
Gill et al., 2019	Cross-sectional study	Canada	128	92	46.32 ± 8.23	33/95	HADS-A ≥ 8
Hanna and Strober, 2020	Cross-sectional study	USA	183	173	44.09 ± 9.51	19/173	STAI
Henry et al., 2019	Cross-sectional study	France	110	70	44.93 ± 12.7	34/76	HADS-A ≥ 8
Jones et al., 2012	Cross-sectional study	UK	4617	2849	50.9 ± 11.5	1355/3253	HADS-A ≥ 8
Karimi et al., 2020	Cross-sectional study	Iran	87	NA	35.5 ± 9.2	26/61	DASS-21 anxiety ≥ 8
Korostil and Feinstein, 2007	Cross-sectional study	Canada	140	78	43.9 ± 10.7	36/104	HADS-A ≥ 10
Kotan et al., 2019	Cross-sectional study	Turkey	227	158	37.0 ± 9.9	64/163	HADS-A
Leonavicius and Adomaitiene, 2013	Cross-sectional study	Lithuania	312	NA	42.01 ± 12.48	116/196	HADS-A
Marck et al., 2016	Cross-sectional study	USA, Canada, Australia, New Zealand, Europe, and others	2399	1472	45.5 ± 10.6	407/1892	SCQ
Marrie et al., 2018	Cross-sectional study	Canada	859	621	48.7 ± 11.3	213/646	HADS-A ≥ 8
Nicholl et al., 2001	Cross-sectional study	UK	96	11	48.7 ± 8.9	11/72	BAI, HADS-A
Noy et al., 1995	Cross-sectional study	Israel	20	NA	41.8 ± 9.0	5/15	HAS ≥ 18
Orr et al., 2018	Cross-sectional study	Canada	251	181	50.9 ± 12.9	47/204	HADS-A ≥ 8
Pham et al., 2018	Cross-sectional study	Canada	244	162	49.5 ± 11.6	99/178	HADS-A ≥ 8
Podda et al., 2020	Cross-sectional study	Italy	608	263	57.9 ± 12.5	200/408	HADS-A ≥ 8
Poder et al., 2009	Cross-sectional study	Canada	245	172	46.1 ± 10.6	45/200	HADS-A ≥ 11
Ramezani et al., 2021	Cross-sectional study	Iran	410	NA	38.60 ± 10.35	84/326	HADS-A ≥ 8
Reyes et al., 2020	Cross-sectional study	UK	236	180	43.5 ± 12.6	68/168	HADS-A ≥ 8
Schiess et al., 2019	Cross-sectional study	USA	416	329	36.6 ± 10.6	148/268	ICD-9
Suh et al., 2010	Cross-sectional study	USA	96	91	42.8 ± 10.2	21/75	HADS-A ≥ 8
Terrill et al., 2015	Cross-sectional study	USA	513	287	51.4 ± 10.9	94/419	GAD-7 ≥ 8
Uguz et al., 2008	Cross-sectional study	Turkey	74	74	34.57 ± 11.93	24/50	SCID-I
van der Hiele et al., 2012	Cross-sectional study	Netherlands	715	251	48.3 ± 10.4	185/530	HADS-A ≥ 8
Wallis et al., 2020	Cross-sectional study	Netherlands	119	79	47.8 ± 9.4	44/75	HADS-A ≥ 8
Zanghì et al., 2020	Cross-sectional study	Italy	432	432	40.4 ± 12.4	155/277	DASS-21 ≥ 8
Panda et al., 2018	Cross-sectional study	India	90	83	38.070 ± 10.470	36/54	HADS-A ≥ 8

RR patients, relapsing and remitting MS patients; M, male; F, female; BAI, Beck Anxiety Inventory; DASS-21, Depression Anxiety and Stress Scale-21; HADS-A, anxiety subscale of the Hospital Anxiety and Depression Scale; ICD-9, International Classification of Diseases, Ninth Revision; HAS, Hamilton Anxiety Scale; SCID-I, Structured Clinical Interview for DSM-IV; SCQ, Self-administered Comorbidity Questionnaire; STAI, State trait anxiety inventory; NA, not available.

1.2 Studies were cross-sectional studies or cohort studies.

2. Exclusion criteria
  2.1 Studies not involving humans, case reports, reviews, guidelines, protocols, commentaries, letters, or abstracts;
  2.2 Studies with insufficient or unavailable data;
  2.3 Duplicate studies;
  2.4 Risk factors involving the study of healthy subjects instead of merely MS patients;
  2.5 Risk factors discussed in fewer than two studies;
  2.6 Non-English articles without English abstracts.

### Study selection

To determine eligibility, two reviewers (XYZ and YS) independently screened all titles and abstracts using Endnote X10 software after the removal of duplicates. An independent third researcher (LY) resolved all divergences between the two reviewers. Subsequently, to determine whether these full texts met the eligibility criteria, two reviewers (XYZ and YS) screened the included full texts of potential interest.

### Data collection process

In this study, two researchers (XYZ and YS) extracted data using preformulated forms. A third researcher (LY) double-checked their results and resolved any disagreements. Supplementary data concerning risk factors were also screened by the researcher (XYZ). For dichotomization, we requested both numbers of patients with anxiety and those without it for each subgroup. And outcomes that could not be dichotomized were eliminated from our analyses.

### Quality assessment

**FIGURE 1:**
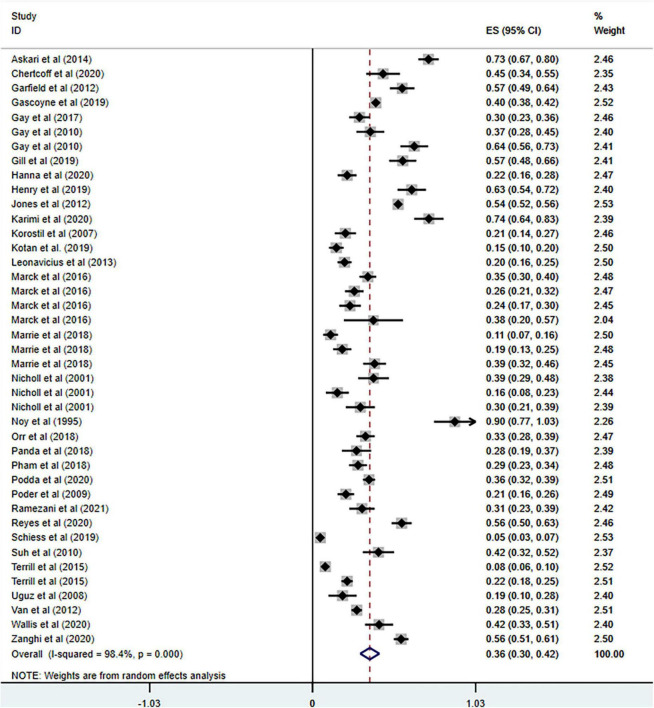
Forest plot of meta-analysis on prevalence of anxiety in MS patients.

Two researchers (XYZ and ZW) evaluated the methodological quality using the guidance from the Agency for Healthcare Research and Quality (AHRQ) Evidence-based Practice Center (Berkman et al., 2008) for the included studies (Figure 1). Research with a total score of >7 out of 11 was considered high-quality (Berkman et al., 2008). Whenever there was a disagreement, it was resolved by consensus.

### Outcome measures

Studies with multiple anxiety evaluation methods were entered as individual study samples. Weighted mean differences (WMDs) were calculated for continuous data, and odds ratios (ORs) were calculated for dichotomous data. And 95% confidence intervals were provided for both WMDs and ORs. A primary outcome measure was anxiety prevalence and ORs with 95% confidence intervals (CIs). The sample sizes or specific values of the subgroups of anxiety and non-anxiety were collected to calculate the WMDs or ORs of each risk factor separately by using the Stata 15.0 (version 15.0, StataCorp, College Station, TX, USA). Sensitivity analyses were run to exclude studies with a high risk of bias.

### Statistical analysis

Data analysis was conducted by XYZ, XC, and XJZ. Fixed effects models were used at the beginning of the analysis. *I*^2^ was assessed using the method proposed by Higgins et al. (2003). In cases where *I*^2^ ≤ 50%, it was determined that there was no obvious heterogeneity among the studies included, and a fixed effect model was applied. Otherwise, *I*^2^ > 50% indicated high heterogeneity (Higgins et al., 2003). In this case, random effect models would be used to calculate the effect size, and a sensitivity analysis and subgroup analysis were conducted to clarify the underlying systematic differences and reduce the substantial heterogeneity. Countries of the studies and measurement tools used in the studies were taken into account for subgroup analyses. In order to compare the significance of the heterogeneity among studies, Chi-squared (χ^2^) tests were conducted. And a conventional *p*-value of 0.05 was used as the cut-off for determining the significance of the heterogeneity.

## Results

### Study selection

**FIGURE 2:**
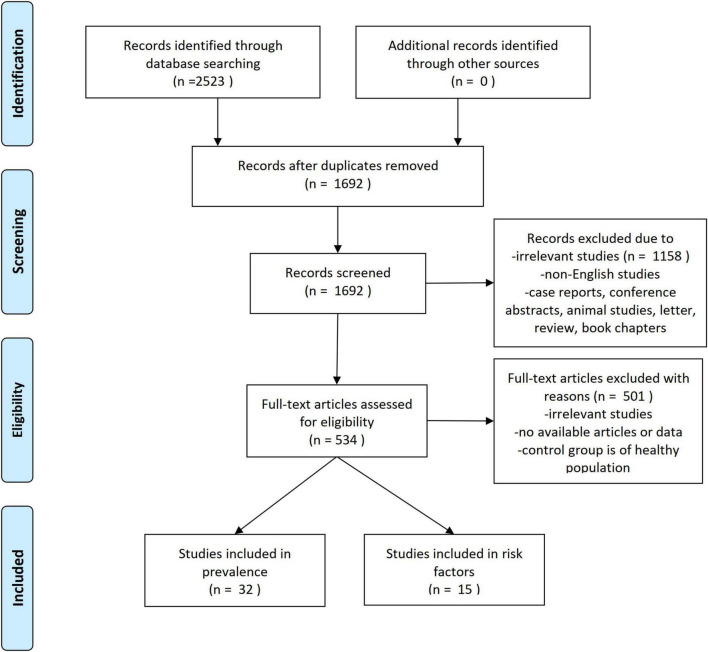
Selection process for the studies included in the meta-analysis.

Through systematic search, 2,523 articles were found. After excluding duplicate papers and irrelevant articles, 534 potentially eligible studies remained. Finally, 32 articles (Noy et al., 1995; Nicholl et al., 2001; Korostil and Feinstein, 2007; Uguz et al., 2008; Poder et al., 2009; Gay et al., 2010, 2017; Suh et al., 2010; Garfield and Lincoln, 2012; Jones et al., 2012; van der Hiele et al., 2012; Leonavicius and Adomaitiene, 2013; Askari et al., 2014; Terrill et al., 2015; Marck et al., 2016; Marrie et al., 2018; Orr et al., 2018; Panda et al., 2018; Pham et al., 2018; Gascoyne et al., 2019; Gill et al., 2019; Henry et al., 2019; Kotan et al., 2019; Schiess et al., 2019; Chertcoff et al., 2020; Hanna and Strober, 2020; Karimi et al., 2020; Podda et al., 2020; Reyes et al., 2020; Wallis et al., 2020; Zanghì et al., 2020; Ramezani et al., 2021) with 15,853 participants were found to be eligible in the analyses, as shown in Table 1. The flow diagram of the search and study selection process was shown in Figure 2.

### Study characteristics

Among the included studies, sample sizes ranged from 20 (Noy et al., 1995) to 4,617 (Jones et al., 2012). Table 1 summarized the clinical characteristics of MS patients enrolled in the included studies. As displayed in Table 1, most studies were cross-sectional, and most of them were conducted in European and American countries including one multi-countries study, with seven studies conducted in Canada, five in the USA and four in the UK. Twenty-three studies assessed anxiety using the anxiety subscale of the Hospital Anxiety and Depression Scale (HADS-A) as a measurement tool. One study diagnosed anxiety according to 9th Revision of the International Classification of Diseases (ICD-9) and another was based on Structured Clinical Interview for DSM-IV (SCID-I) (First et al., 2007). As for the analysis of risk factors of anxiety in MS patients, only fifteen studies were eligible for further investigation.

### Quality of studies

Agency for Healthcare Research and Quality checklists were used to assess the quality of cross-sectional studies. The checklist contains 11 items with the options of “Yes,” “No,” or “unclear.” For each item, the answers of “no” or “unclear” were recorded as “0” and the answer of “yes” was marked as “1” (Landeiro et al., 2011). Based on the total score, the included studies were divided into the following categories: good (8–11), average (4–7), and poor (0–3).

Based on the AHRQ checklist, the included studies’ methodological quality was strong with a mean score of 9.78 ± 1.16 out of 11 and a score range of 7 to 11 out of 11 (see Table 2). The main weakness of the included studies was that quite a few studies did not mention whether the subjects were consecutive if not population-based.

**TABLE 2:** Agency for Healthcare Research and Quality assessment of included studies.

References	Item 1	Item 2	Item 3	Item 4	Item 5	Item 6	Item 7	Item 8	Item 9	Item 10	Item 11	Total
Askari et al., 2014	1	1	1	Unclear	1	1	1	1	1	1	1	10
Chertcoff et al., 2020	1	1	1	1	1	1	1	1	1	1	1	11
Garfield and Lincoln, 2012	1	1	1	Unclear	1	1	1	1	1	1	1	10
Gascoyne et al., 2019	1	1	1	1	1	1	1	1	1	1	1	11
Gay et al., 2017	1	0	1	Unclear	1	1	1	1	1	1	1	9
Gay et al., 2010	1	0	0	Unclear	1	1	1	1	1	1	1	8
Gill et al., 2019	1	1	1	0	1	1	1	1	1	1	1	10
Hanna and Strober, 2020	1	1	1	1	1	1	1	1	1	1	1	11
Henry et al., 2019	1	1	0	Unclear	1	1	1	1	1	1	1	9
Jones et al., 2012	1	0	1	1	1	0	0	1	0	1	1	7
Karimi et al., 2020	1	1	1	1	1	1	1	1	1	1	1	11
Korostil and Feinstein, 2007	1	1	0	1	1	1	1	1	1	1	1	10
Kotan et al., 2019	1	1	0	Unclear	1	0	1	1	1	1	1	8
Leonavicius and Adomaitiene, 2013	1	0	1	Unclear	0	0	1	1	1	1	1	7
Marck et al., 2016	1	0	1	1	0	1	1	1	1	1	1	9
Marrie et al., 2018	1	0	1	1	1	1	1	1	1	1	1	10
Nicholl et al., 2001	1	0	1	1	1	1	1	1	1	1	1	10
Noy et al., 1995	1	1	0	Unclear	1	1	1	1	1	1	1	9
Orr et al., 2018	1	1	0	1	1	1	1	1	1	1	1	9
Pham et al., 2018	1	1	1	Unclear	1	1	1	1	1	1	1	10
Podda et al., 2020	1	1	1	1	1	1	1	1	1	1	1	11
Poder et al., 2009	1	1	1	1	1	1	1	1	1	1	1	11
Ramezani et al., 2021	1	0	1	Unclear	1	1	1	1	1	1	1	9
Reyes et al., 2020	1	1	1	Unclear	1	1	1	1	1	1	1	10
Schiess et al., 2019	1	1	1	1	1	1	1	1	1	1	1	11
Suh et al., 2010	1	1	0	Unclear	1	1	1	1	1	1	1	9
Terrill et al., 2015	1	1	1	Unclear	1	1	1	1	1	1	1	10
Uguz et al., 2008	1	1	1	1	1	1	1	1	1	1	1	11
van der Hiele et al., 2012	1	1	1	1	1	1	1	1	1	1	1	11
Wallis et al., 2020	1	1	1	1	1	1	1	1	1	1	1	11
Zanghì et al., 2020	1	1	1	Unclear	1	1	1	1	1	1	1	10
Panda et al., 2018	1	1	0	1	1	1	1	1	1	1	1	10

### Prevalence of anxiety in MS

**FIGURE 3:**
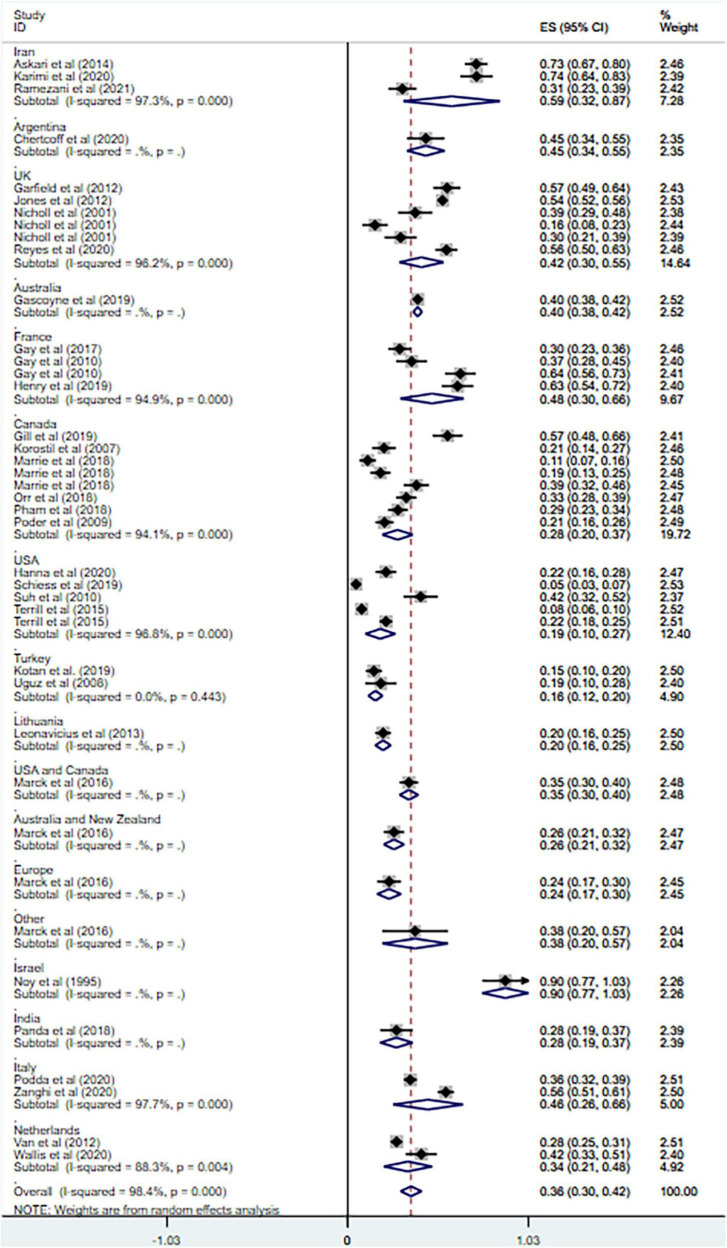
Forest plot of subgroup analysis on changes of prevalence of anxiety in MS based on country.

**FIGURE 4:**
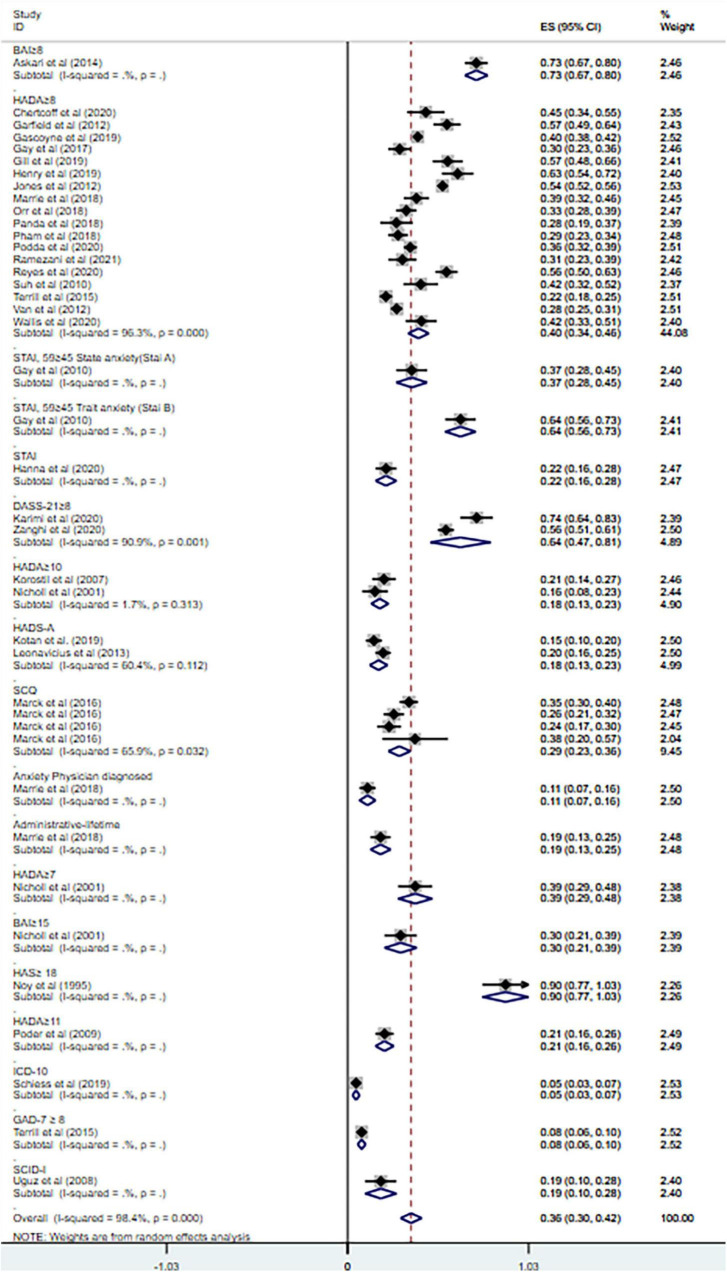
Forest plot of subgroup analysis on changes of prevalence of anxiety in MS based on measurement tool.

**FIGURE 5:**
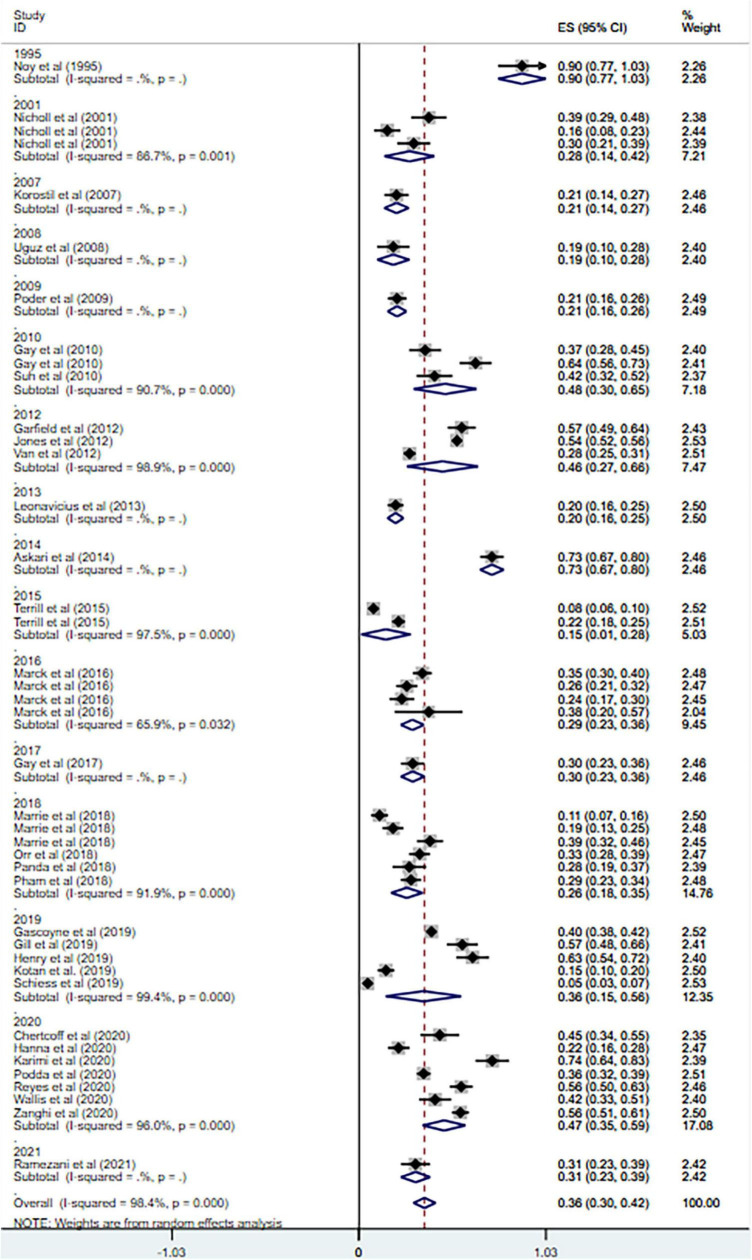
Forest plot of subgroup analysis on changes of prevalence of anxiety in MS based on publication year.

**FIGURE 6:**
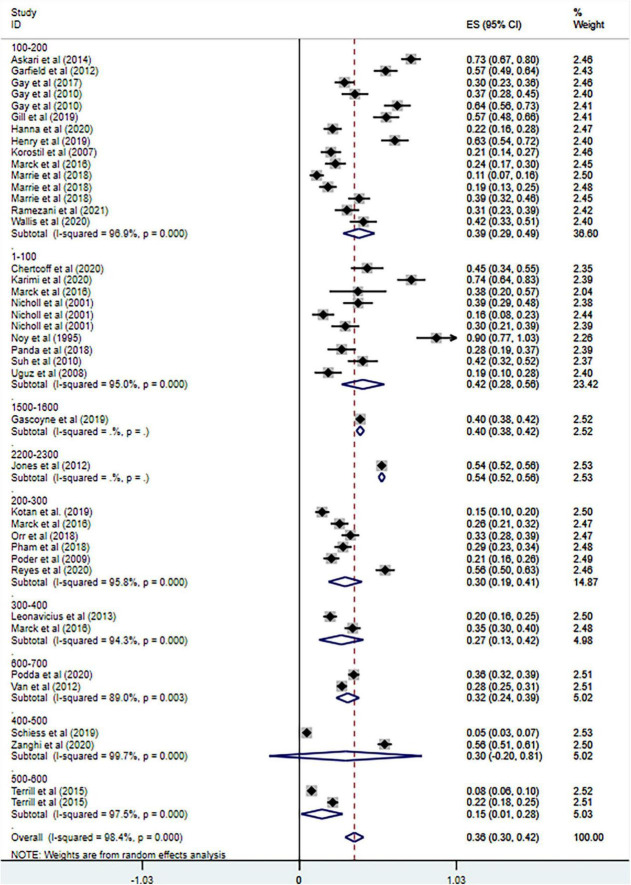
Forest plot of subgroup analysis on changes of prevalence of anxiety in MS based on total sample size.

Anxiety is prevalent in MS at 36% (95% CI = [0.30–0.42], *I*^2^ = 98.4%, *p* < 0.001; 32 studies). And high heterogeneity was still observed after subgroup analyses based on geographical country, measurement tool, publication year and sample size (see Figures 3–6).

### Risk factors for anxiety in MS

Fifteen studies reported more than 50 risk factors for the development of anxiety in MS, as summarized in Tables 3, 4. The risk factors presented in at least two studies are as follows: gender (female or not), prior psychiatric history (past history of psychiatric disorders or psychiatric comorbidity), age at survey, comorbidity with depression, family history of psychiatric disorders, family status (living together or living alone), employment status (currently working or not), education level, marriage, disease course (type of MS), disease duration (time since diagnosis in years), lack of disease-modifying therapy (DMT) and Expanded Disability Status Scale (EDSS) scores at baseline (T0). Meta analyses were conducted for the risk factors mentioned in more than one study. Supplementary figures can be found in Supplementary Appendix 2.

**TABLE 3:** Factors correlated with anxiety status in MS patients.

References	Factor
Ramezani et al., 2021	Age at survey, age at the onset of MS, history of psychiatric disorders, history of taking psychiatric medication, history of COVID-19 in family, education, occupation, immunomodulation, immunosuppressor
Podda et al., 2020	Age at survey, gender, level of education (university, high school, primary school), disease duration, disease course, EDSS at T0, MoCA at T0, HADS-D at T0, HADS-A at T0, FIS at T0
Karimi et al., 2020	Gender, family history, history of the disease, smoking, physical activity, mental status, occupation
Hanna and Strober, 2020	Age, gender, education, disease course, disease duration
Chertcoff et al., 2020	Gender (female),age, education (in years), marital status, currently smoking, prior psychiatric history, family history of psychiatric disorders, time since diagnosis, disease subtype, current DMT, EDSS, depression, COOP/WONCA score
Marrie et al., 2018	Gender, race, education, age, disease duration, EDSS score, clinical score, clinical course, smoking
Marrie et al., 2018	Gender, age, socioeconomic status, region
Terrill et al., 2015	Gender, race, employment status, married, MS type, age, MS duration, PHQ-9 score
Askari et al., 2014	BDI, EDSS, age, gender, disease type
Leonavicius and Adomaitiene, 2013	Age, family status, residence, MS duration
Jones et al., 2012	Gender, MS type, depression level
Garfield and Lincoln, 2012	Age, years since diagnosis, months since last relapse, HADS Depression, GHQ-12, GNDS score, MSSS, MHLC, PSS
Korostil and Feinstein, 2007	Cognitively impaired, depression, disease course, disease duration, EDSS, education, employed, family history of mental illness, gender, HAD, living alone, major depression, married, SSSI, substance abuse, suicidal intent, suicide attempt, taking disease modifying treatments
Zanghì et al., 2020	Marital status, age, psychiatric comorbidity, EDSS score, disease duration, number of relapse in the year before, starting or changing DMT in the last 12 months, line of DMT actually taken
Gascoyne et al., 2019	Age, gender, type of MS at onset, type of MS now, PDDS, prevalent fatigue, years since symptom onset, employed, socio-economic status, rurality
Pham et al., 2018	Gender, education, employment, smoking, alcohol, illicit drug use, disease type, disease modifying medication, adverse medication side effects, EDSS score, depression

MS, multiple sclerosis; COVID-19, Coronavrus Disease 2019; EDSS, Expanded Disability Status Scale; MoCA, Montreal Cognitive Assessment; HAD, Hospital Anxiety and Depression; HADS-D, Hospital Depression and Anxiety Scale Depression subscale; HADS Depression, Hospital Depression and Anxiety Scale Depression subscale; HADS-A, Hospital Depression and Anxiety Scale Anxiety subscale; FIS, Fatigue Impact Scale; T0, at baseline; PHQ-9, Patient Health Questionnaire-Nine; DMT, disease modifying treatments; BDI, Beck Depression Inventory; GHQ-12, General Health Questionnaire-12; GNDS score, General Neuropsychological Deficit Score; MSSS, Multiple Sclerosis Severity Score; MHLC, Multidimensional Health Locus of Control scale; PSS, Perceived Stress Scale; SSSI, Social Stress and Support Interview; PDDS, Patient Determined Disease Steps.

**TABLE 4:** Comparisons on risk factors for anxiety in patients with MS.

Risk factor	Effect size, confidence interval, *p*-value, *I*^2^	Total number of studies included
Age at survey (Supplementary Figure 1)	WMD 0.96, 95% CI = [0.86–1.06], *p* < 0.001, *I*^2^ = 43.8%	6
Gender (Supplementary Figure 2)	OR 1.78, 95% CI = [1.38–2.30], *p* < 0.001, *I*^2^ = 0	8
Depression (Supplementary Figure 3)	OR 7.89, 95% CI = [3.71–16.81], *p* < 0.001, *I*^2^ = 0	3
Marriage status (Supplementary Figure 4)	OR 0.95, 95% CI = [0.62–1.44], *p* = 0.794, *I*^2^ = 40.7%	3
Employment status (Supplementary Figure 5)	OR 0.87, 95% CI = [0.6–1.24], *p* = 0.434, *I*^2^ = 27.5%	3
Education levels (Supplementary Figure 6)	OR 1.28, 95% CI = [0.52–3.15], *p* = 0.830, *I*^2^ = 85.5%	3
Years of education (Supplementary Figure 7)	WMD −0.03, 95% CI = [−0.32 to 0.26], *p* = 0.830, *I*^2^ = 7.9%	2
Disease duration (Supplementary Figure 8)	WMD 0, 95%, CI = [−0.21–0.22], *p* = 0.966, *I*^2^ = 0	4
Disease course (Supplementary Figure 9)	OR 1.50, 95% CI = [1.14–1.99], *p* = 0.004, *I*^2^ = 53.5%	7
Past psychiatric history (Supplementary Figure 10)	OR 2.83, 95% CI = [1.74–4.59], *p* < 0.001, *I*^2^ = 0	2
Living together (Supplementary Figure 11)	OR 2.83, 95% CI = [1.74–4.59], *p* < 0.001, *I*^2^ = 0	2
Baseline EDSS scores (Supplementary Figure 12)	WMD 0.84, 95% CI = [0.48–1.21], *p* < 0.001, *I*^2^ = 62.2%	2
Lack of DMT therapy (Supplementary Figure 13)	OR 2.33, 95% CI = [1.29–4.21], *p* < 0.001, *I*^2^ = 77.8%	2
Family psychiatric history (Supplementary Figure 14)	OR 2.42, 95% CI = [1.56–3.75], *p* = 0.158, *I*^2^ = 0	2

#### Age at survey

Age at survey was discussed in six studies and one study (Podda et al., 2020) with a high risk of bias was withdrawn. Fixed effect model analysis showed that there was no correlation between age and anxiety (WMD 0.96, 95% CI = [0.86–1.06], *p* < 0.001, *I*^2^ = 43.8%) (see Supplementary Figure 1).

#### Gender

Data from eight eligible studies were combined and a sensitivity analysis was carried out with a high-risk bias study (Jones et al., 2012) withdrawn. The analysis results showed that female MS patients were 1.78 times more likely to have anxiety than male MS patients (OR 1.78, 95% CI = [1.38–2.30], *p* < 0.001, *I*^2^ = 0) (see Supplementary Figure 2).

#### Depression

Depression was investigated in three studies. After excluding one study (Jones et al., 2012) with a high risk of bias after sensitivity analysis, the heterogeneity remained zero (*I*^2^ = 0, *p* = 0.560) with an increase of the intergroup difference. Our result showed that in MS patients who had depression, anxiety was 7.89 times more likely to develop than in those without depression (OR 7.89, 95% CI = [3.71–16.81], *p* < 0.001, *I*^2^ = 0) (see Supplementary Figure 3).

#### Marriage status

The combined analysis of marriage status in three studies showed that there was no significant relationship between marriage status and anxiety (OR 0.95, 95% CI = [0.62–1.44], *p* = 0.794) without heterogeneity (*I*^2^ = 40.7%) (see Supplementary Figure 4).

#### Employment status

The results of three studies concerning employment status were pooled, and no significant association was found between the development of anxiety and employment (OR 0.87, 95% CI = [0.6–1.24], *p* = 0.434) without heterogeneity (*I*^2^ = 27.5%) (see Supplementary Figure 5).

#### Education

Education was reported in five studies, three of which provided binary variables (education levels) and two provided continuous variables (years of education). The analysis of binary variables showed no significant association between education and the development of anxiety (OR 1.28, 95% CI = [0.52–3.15], *p* = 0.830) (see Supplementary Figure 6) with high heterogeneity (*I*^2^ = 85.5%), while the analysis of continuous variables also showed no significant association between education and anxiety (WMD −0.03, 95% CI = [−0.32 to 0.26], *p* = 0.830) and the between-group heterogeneity was small (*I*^2^ = 7.9%) (see Supplementary Figure 7). Sensitivity analysis of binary variables did not find the cause of heterogeneity.

#### Disease duration

Four studies reported the effect of disease duration on the development of anxiety in MS patients. After eliminating a study (Jones et al., 2012) with a high risk of bias, the result showed no significant relationship between disease duration and anxiety and the heterogeneity between studies dropped to zero (*I*^2^ = 0) with a decreased intergroup difference (WMD 0 95%, CI = [−0.21 to 0.22], *p* = 0.966) (see Supplementary Figure 8).

#### Disease course

Seven studies reported disease course (types of MS) as a risk factor. After excluding one study (Jones et al., 2012) with a high risk of bias after sensitivity analysis, results demonstrated that RRMS patients were 1.50 times more likely to develop anxiety than those with other types of MS (OR 1.50, 95% CI = [1.14–1.99], *p* = 0.004, *I*^2^ = 53.5%) (see Supplementary Figure 9).

#### Others

The remaining risk factors were discussed in only two studies: past psychiatric history, family psychiatric history, family status, DMT therapy (including interferon beta, glatiramer acetate, fingolimod, dimethyl fumarate, teriflunomide, alemtuzumab, and natalizumab), and baseline EDSS scores. And the results showed that MS patients with prior psychiatric history were 2.83 times more likely to have anxiety (OR 2.83, 95% CI = [1.74–4.59], *p* < 0.001) (see Supplementary Figure 10). And those living with other family members were more anxious than those living alone (OR 2.83, 95% CI = [1.74–4.59], *p* < 0.001) (see Supplementary Figure 11). But no significant effect of family history of psychiatric disorders on anxiety was found (OR 2.42, 95% CI = [1.56–3.75], *p* = 0.158) (see Supplementary Figure 12). No significant heterogeneity was perceived for the above three risk factors (*I*^2^ = 0). For EDSS scores at baseline, those with higher scores were probably less likely to have anxiety (WMD 0.84, 95% CI = [0.48–1.21], *p* < 0.001) with heterogeneity (*I*^2^ = 62.2%) (see Supplementary Figure 13). And among patients who did not take DMT medication, anxiety was 2.33 times more common than among those who did (OR 2.33, 95% CI = [1.29–4.21], *p* < 0.001) with a heterogeneity of *I*^2^ = 77.8% (see Supplementary Figure 14). Other risk factors that were discussed only in one study included COVID-19, comorbidity, races, physical exercise, smoking, drinking, illicit drug use, diet, residency, social status, cognition decline, fatigue severity, etc.

### Publication bias

**FIGURE 7:**
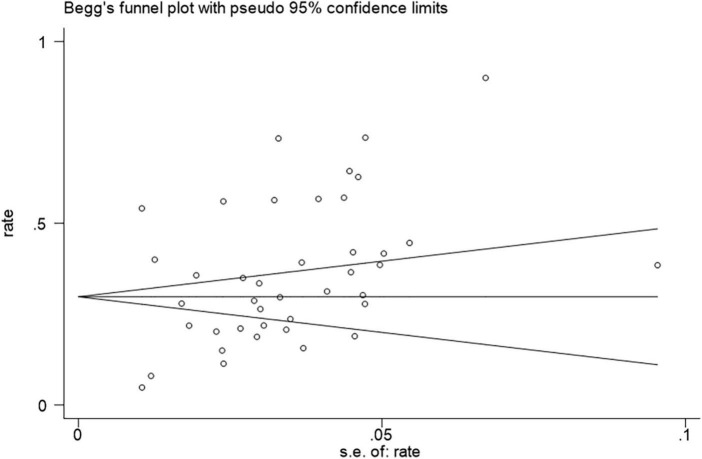
Funnel plot of meta-analysis on prevalence of anxiety in MS patients.

We evaluated publication bias on the prevalence of anxiety in MS through Egger’s and Begg’s tests with the use of Stata15. The *p*-values of Egger’s and Begg’s tests were mixed (Begg’s test 0.011, Egger’s test 0.121), which appeared that publication bias may exist in our study. Begg’s funnel plot for publication bias is shown in Figure 7.

## Discussion

The current study is the first meta-analysis to investigate both the prevalence and the risk factors in the development of anxiety in MS patients. With a relatively large sample of 15,853 patients in 32 studies conducted in 15 countries, the latest data as of May 2021 on the prevalence of anxiety in MS and comparing anxiety and non-anxiety populations was collected to examine the prevalence and risk factors of anxiety in MS patients.

Overall, the prevalence of anxiety among MS patients was 36% (95% CI = [0.30–0.42]) according to our results, which is in line with previous studies with the range of 22.1% (Boeschoten et al., 2017) to 55% (Hauer et al., 2021). However, the heterogeneity of our study of prevalence was extremely high, and thus subgroup analyses were conducted based on country, measurement tools, publication year, and sample size. The subgroup analysis based on country showed the heterogeneity sharply decreased in the analysis of Turkey, but did not change much in other countries. And the subgroup analysis based on measurement tools resulted in the decrease of heterogeneity in the combined HADA ≥ 10 group and HADS-A group (cut-off values not mentioned). As for the analysis of other measurement tools, the heterogeneity remained high. Little change was found after subgroup analysis based on publication year or sample size. The heterogeneity of previous review on anxiety in MS was also high and the variations still presented after subgroup analyses (Boeschoten et al., 2017), which is consistent with our results.

Thus, it seems impossible to provide an exact picture of the prevalence of anxiety among patients with MS considering the significant heterogeneity of the pooled estimates, and various reasons may account for the discrepancies in the prevalence. First of all, the prevalence of MS varies across ethnic groups, regions and countries (Pugliatti et al., 2002) and the diagnostic criteria of MS have been updated over the years (Polman et al., 2005, 2011; Thompson et al., 2018), resulting in differences in the sample population characteristics in the included studies. Secondly, the evaluation of anxiety disorder relies on measurement tools and different cut-off values and is a lack of consolidated standards (Brooks and Kutcher, 2003), and measurement tools may not be objective enough for the diagnosis of anxiety. Thirdly, patients with the same criteria of MS diagnosis may differ in the disease courses, duration, disability level, family, and economic status and past medical history, which would certainly greatly affect anxiety development. And because of that, we also discussed the risk factors of anxiety in our review.

Multiple sclerosis anxiety risk factors were another objective of this review. The following factors were investigated in our study: gender (female or not), prior psychiatric history, age at survey, comorbidity with depression, family history of psychiatric disorders, family status (living together or not), employment status, education, marriage, disease course (disease subtype), disease duration, treatment compliance, and EDSS scores at baseline. Among them, the results showed that age at survey, female, family status, prior psychiatric history, comorbidity with depression, usage of MS medication, and baseline EDSS were significantly correlated with the development of anxiety in patients with MS.

As shown in Table 4, age and gender are the most commonly discussed risk factors. Our study showed that female MS patients were prone to have an increased rate of anxiety. According to studies, MS has a female predominance, and anxiety is highly associated with women (Koch-Henriksen and Sørensen, 2010; Sellner et al., 2011). And a multicenter survey of 3,142 adults showed the prevalence rate of anxiety disorders was higher in female populations, but dropped in adults aged 75–84 compared with 65–74 (Canuto et al., 2018). Potential factors contributing to gender discrepancy include anxiety sensitivity (Norr et al., 2015), stress coping style (Altemus et al., 2014; Kiely et al., 2019) or fluctuations in exposure to reproductive hormones and peptides during the menstrual cycle (Altemus et al., 2014; Kiely et al., 2019). However, little data are available on the relationship between age and anxiety. A recent review showed that anxiety symptoms may be more difficult to elicit in the elderly, as they are less accurate in identifying anxiety symptoms and tend to attribute them to physical illness rather than anxiety itself. Another survey indicated that the age-sex pattern for anxiety in the general population was only observed during fertile periods, while the risk for new cases became similar for both genders after menopause (Faravelli et al., 2013). Few articles have addressed the effects of aging on the development of anxiety in MS. Our review found the age disparity, but could not show the prevalence rate among the different age groups of a specific gender owing to the lack of original data from included studies.

Prior psychiatric history and comorbidity of depression were also found to be significantly associated with the prevalence of anxiety in MS patients. Pre-existing mental disorders will increase the prevalence of anxiety after the diagnosis of MS, and anxiety, depression, and fatigue tend to cluster together (Wood et al., 2013; Demyttenaere and Heirman, 2020). The incorporation of phenomenological, psychopathological and evolutionary concepts may be helpful in understanding the articulation between anxiety and depression. From a phenomenological approach, both the diagnostic criteria from classification systems and items of measurement tools for anxiety overlap significantly with those of depression (Tyrer, 2018; Demyttenaere and Heirman, 2020; Konac et al., 2021). From the perspective of pathophysiological mechanism, anxiety and depression are both characterized by neuroendocrine disorders (Boyer, 2000). Historically, the monoamine hypothesis has dominated anxiety and depression research and treatment. While the research into neuropeptide systems throws greater views on the understanding of the pathogenesis of the comorbidity (Alldredge, 2010). Recent research has also focused on stress on the signal transduction on the pathogenesis of anxiety and depression. Stress-induced pathogenic stimuli activate endothelial and perivascular microglia, and mediate perivascular neurons and peripheral astrocytes, thereby controlling the formation of pre-inflammatory and anti-inflammatory phenotypes of astrocytes and microglia in the blood brain barrier (Welcome, 2020). The above mechanisms provide insight into prevention and treatment of anxiety and depression for MS patients.

No significant associations between anxiety and the disease duration or disease course of MS patients were found in our study. Nevertheless, our results showed that patients not taking MS medication and those with a higher baseline EDSS tended to have a higher prevalence rate of anxiety. That may imply MS patients with severe symptoms may be more likely to experience anxiety, and medication treatment may alleviate anxiety symptoms to some degree. As previous surveys suggested that anxiety is related to physical severity (Lester et al., 2007), interventions for self-management may improve anxiety and QOL for MS patients (Kidd et al., 2017). Moreover, patients with less changes in severity of symptoms may have less variation in work productivity as well (Bessing et al., 2021). The above findings suggest that MS patients should be more active in the intervention of the disease, which may decrease the prevalence of psychiatric complications and improve their QOL.

Our review also found that family status may probably affect the development of anxiety in MS, but no significant correlations was observed between anxiety and risk factors including employment status, education, and family history of psychiatric in MS patients. In terms of family relationships, living together with family members may increase the prevalence rate of anxiety when compared with living alone. A survey of 2,057 medical students showed that anxiety symptoms had highly significant correlations with family status, social support, and coping style (Shao et al., 2020). And people who had bad relationships with their lovers, classmates or friends scored higher on anxiety tests (Shao et al., 2020), since life-threatening diseases may cause much more severe stress that can lead to marital discord, separation, or divorce (Hakim et al., 2000). Patients who lived together with family members may experience higher level of anxiety due to deteriorated couple relationships and a greater probability of divorce (Glantz et al., 2009).

Compared with depression, anxiety receives less attention from clinicians or researchers, whereas anxiety often accompanies depression and can decrease patients’ and caregivers’ QOL. Thus, it is necessary to focus on the prevalence and risk factors of anxiety in chronic diseases like MS. Our study demonstrated the prevalence of anxiety in patients with MS and the potential risk factors including age at survey, female, family status, prior psychiatric history, comorbidity with depression, treatment compliance, and baseline EDSS. However, as the reliability of current results is limited by small sample size, outcome measures and cross-sectional designs, our results should be interpreted cautiously and need further validation with more qualitative research. In the future, a more accurate classification and more detailed description of the target population should be provided. Besides, future detection of anxiety should not solely rely on measurement tools, but also on objective evaluation tools or diagnostic markers such as neuroimaging, neurophysiology, and biochemistry (Koch-Henriksen and Sørensen, 2010). Furthermore, longitudinal designs are needed to develop a deeper understanding of the associations between significant risk factors and anxiety in MS patients.

## Conclusion

In this meta-analysis, we summarized the prevalence of anxiety in MS and analyzed common significant risk factors that contribute to anxiety development in MS patients. Our study estimated that 36% of MS patients suffer from anxiety. And we found that age at survey, female, living together, past psychiatric history, depression, compliance with MS medications, RRMS, and baseline EDSS are significant risk factors for anxiety in MS patients. Our results help serve as a reminder to both patients and physicians of various risk factors that contribute to anxiety, and highlight the necessity of monitoring patients with modifiable risk factors to prevent further costs and aggravation.

## Data availability statement

The original contributions presented in this study are included in the article/Supplementary material, further inquiries can be directed to the corresponding author.

## Author contributions

XYZ involved in the study’s conceptualization and design, and wrote the original draft of the work. XYZ and YS extracted the data using pre-made forms. XYZ, XC, and XJZ carried out the statistical analysis. All authors reviewed, revised, and approved the submitted version of the work.
