# Supplementary material for: The prevalence and risk factors of anxiety in multiple sclerosis: A systematic review and meta-analysis
Source: Front Neurosci. 2023 Apr 17;17:1120541. doi: 10.3389/fnins.2023.1120541 (PMC10149809; doi:10.3389/fnins.2023.1120541)
Supplement: Supplementary file 1 [file Data_Sheet_1.pdf]

Appendix 1. Overview of the applied search terms in searching of databases

Pubmed

Search number Query Sort By Filters Search Details Results Time

1 "Multiple Sclerosis"[Mesh] Most Recent "Multiple Sclerosis"[MeSH Terms] 61,609 2:46:49

2 (((((Multiple Sclerosis[Title/Abstract]) OR (Sclerosis, Multiple[Title/Abstract])) OR (Sclerosis, Disseminated[Title/Abstract])) OR (Disseminated Sclerosis[Title/Abstract])) OR (MS (Multiple Sclerosis)[Title/Abstract])) OR (Multiple Sclerosis, Acute Fulminating[Title/Abstract]) "multiple sclerosis"[Title/Abstract] OR "sclerosis multiple"[Title/Abstract] OR "sclerosis disseminated"[Title/Abstract] OR "disseminated sclerosis"[Title/Abstract] OR (("ms"[Journal] OR "med sci paris"[Journal] OR "ms"[All Fields]) AND ("multiple sclerosis"[MeSH Terms] OR ("Multiple"[All Fields] AND "Sclerosis"[All Fields]) OR "multiple sclerosis"[All Fields])) OR "multiple sclerosis acute fulminating"[Title/Abstract] 82,357 2:49:46

3 ((((((Multiple Sclerosis[Title/Abstract]) OR (Sclerosis, Multiple[Title/Abstract])) OR (Sclerosis, Disseminated[Title/Abstract])) OR (Disseminated Sclerosis[Title/Abstract])) OR (MS (Multiple Sclerosis)[Title/Abstract])) OR (Multiple Sclerosis, Acute Fulminating[Title/Abstract])) OR ("Multiple Sclerosis"[Mesh]) "Multiple Sclerosis"[Title/Abstract] OR "sclerosis multiple"[Title/Abstract] OR "sclerosis disseminated"[Title/Abstract] OR "disseminated sclerosis"[Title/Abstract] OR (("ms"[Journal] OR "med sci paris"[Journal] OR "ms"[All Fields]) AND ("Multiple Sclerosis"[MeSH Terms] OR ("Multiple"[All Fields] AND "Sclerosis"[All Fields]) OR "Multiple Sclerosis"[All Fields])) OR "multiple sclerosis acute fulminating"[Title/Abstract] OR "Multiple Sclerosis"[MeSH Terms] 88,118 2:50:07

4 "Anxiety"[Mesh] Most Recent "Anxiety"[MeSH Terms] 91,652 2:51:06

5 (((((((Anxiety[Title/Abstract]) OR (Angst[Title/Abstract])) OR (Nervousness[Title/Abstract])) OR (Hypervigilance[Title/Abstract])) OR (Anxiousness[Title/Abstract])) OR (Social Anxiety[Title/Abstract])) OR (Anxieties, Social[Title/Abstract])) OR (Social Anxieties[Title/Abstract]) "Anxiety"[Title/Abstract] OR "Angst"[Title/Abstract] OR "Nervousness"[Title/Abstract] OR "Hypervigilance"[Title/Abstract] OR "Anxiousness"[Title/Abstract] OR "social anxiety"[Title/Abstract] OR (("Anxiety"[MeSH Terms] OR "Anxiety"[All Fields] OR "Anxieties"[All Fields] OR "anxiety s"[All Fields]) AND "Social"[Title/Abstract]) OR "social anxieties"[Title/Abstract] 216,693 2:52:32

6 ("Anxiety"[Mesh]) OR ((((((((Anxiety[Title/Abstract]) OR (Angst[Title/Abstract])) OR (Nervousness[Title/Abstract])) OR (Hypervigilance[Title/Abstract])) OR (Anxiousness[Title/Abstract])) OR (Social Anxiety[Title/Abstract])) OR (Anxieties, Social[Title/Abstract])) OR (Social Anxieties[Title/Abstract])) "Anxiety"[MeSH Terms] OR ("Anxiety"[Title/Abstract] OR "Angst"[Title/Abstract] OR

"Nervousness"[Title/Abstract] OR "Hypervigilance"[Title/Abstract] OR "Anxiousness"[Title/Abstract] OR "social anxiety"[Title/Abstract] OR (("Anxiety"[MeSH Terms] OR "Anxiety"[All Fields] OR "Anxieties"[All Fields] OR "anxiety s"[All Fields]) AND "Social"[Title/Abstract]) OR "social anxieties"[Title/Abstract])239,810 2:52:52

7 "Prevalence"[Mesh] Most Recent "Prevalence"[MeSH Terms] 307,801 2:53:36

8 (((((((Prevalence[Title/Abstract]) OR (Prevalences[Title/Abstract])) OR (Period Prevalence[Title/Abstract])) OR (Period Prevalences[Title/Abstract])) OR (Prevalence, Period[Title/Abstract])) OR (Point Prevalence[Title/Abstract])) OR (Point Prevalences[Title/Abstract])) OR (Prevalence, Point[Title/Abstract])

"Prevalence"[Title/Abstract] OR "Prevalences"[Title/Abstract] OR "period prevalence"[Title/Abstract] OR "period prevalences"[Title/Abstract] OR "prevalence period"[Title/Abstract] OR "point prevalence"[Title/Abstract] OR "point prevalences"[Title/Abstract] OR "prevalence point"[Title/Abstract] 675,142 2:55:00

9 ("Prevalence"[Mesh]) OR ((((((((Prevalence[Title/Abstract]) OR (Prevalences[Title/Abstract])) OR (Period Prevalence[Title/Abstract])) OR (Period Prevalences[Title/Abstract])) OR (Prevalence, Period[Title/Abstract])) OR (Point Prevalence[Title/Abstract])) OR (Point Prevalences[Title/Abstract])) OR (Prevalence, Point[Title/Abstract])) "Prevalence"[MeSH Terms] OR "Prevalence"[Title/Abstract] OR "Prevalences"[Title/Abstract] OR "period prevalence"[Title/Abstract] OR "period prevalences"[Title/Abstract] OR "prevalence period"[Title/Abstract] OR "point prevalence"[Title/Abstract] OR "point prevalences"[Title/Abstract] OR "prevalence point"[Title/Abstract] 761,650 2:55:26

10 "Risk Factors"[Mesh]Most Recent "Risk Factors"[MeSH Terms] 867,300 2:56:04

11 ((((((Risk Factors[Title/Abstract]) OR (Factor, Risk[Title/Abstract])) OR (Risk Factor[Title/Abstract])) OR (Health Correlates[Title/Abstract])) OR (Correlates, Health[Title/Abstract])) OR (factor[Title/Abstract])) OR (factors[Title/Abstract])

"risk factors"[Title/Abstract] OR "factor risk"[Title/Abstract] OR "risk factor"[Title/Abstract] OR "health correlates"[Title/Abstract] OR "correlates health"[Title/Abstract] OR "Factor"[Title/Abstract] OR "Factors"[Title/Abstract] 3,583,202 2:57:05

12 ("Risk Factors"[Mesh]) OR (((((((Risk Factors[Title/Abstract]) OR (Factor, Risk[Title/Abstract])) OR (Risk Factor[Title/Abstract])) OR (Health Correlates[Title/Abstract])) OR (Correlates, Health[Title/Abstract])) OR (factor[Title/Abstract])) OR (factors[Title/Abstract])) "Risk Factors"[MeSH Terms] OR "Risk Factors"[Title/Abstract] OR "factor risk"[Title/Abstract] OR "risk factor"[Title/Abstract] OR "health correlates"[Title/Abstract] OR "correlates health"[Title/Abstract] OR "Factor"[Title/Abstract] OR "Factors"[Title/Abstract] 4,030,263 2:57:27

13 (("Prevalence"[Mesh]) OR ((((((((Prevalence[Title/Abstract]) OR (Prevalences[Title/Abstract])) OR (Period Prevalence[Title/Abstract])) OR (Period Prevalences[Title/Abstract])) OR (Prevalence, Period[Title/Abstract])) OR (Point

Prevalence[Title/Abstract])) OR (Point Prevalences[Title/Abstract])) OR (Prevalence, Point[Title/Abstract]))) OR (("Risk Factors"[Mesh]) OR (((((((Risk Factors[Title/Abstract]) OR (Factor, Risk[Title/Abstract])) OR (Risk Factor[Title/Abstract])) OR (Health Correlates[Title/Abstract])) OR (Correlates, Health[Title/Abstract])) OR (factor[Title/Abstract])) OR (factors[Title/Abstract])))

"Prevalence"[MeSH Terms] OR "Prevalence"[Title/Abstract] OR "Prevalences"[Title/Abstract] OR "period prevalence"[Title/Abstract] OR "period prevalences"[Title/Abstract] OR "prevalence period"[Title/Abstract] OR "point prevalence"[Title/Abstract] OR "point prevalences"[Title/Abstract] OR "prevalence point"[Title/Abstract] OR "Risk Factors"[MeSH Terms] OR "Risk Factors"[Title/Abstract] OR "factor risk"[Title/Abstract] OR "risk factor"[Title/Abstract] OR "health correlates"[Title/Abstract] OR "correlates health"[Title/Abstract] OR "Factor"[Title/Abstract] OR "Factors"[Title/Abstract] 4,514,085 2:58:06

14 ((((((((Multiple Sclerosis[Title/Abstract]) OR (Sclerosis, Multiple[Title/Abstract])) OR (Sclerosis, Disseminated[Title/Abstract])) OR (Disseminated Sclerosis[Title/Abstract])) OR (MS (Multiple Sclerosis)[Title/Abstract])) OR (Multiple Sclerosis, Acute Fulminating[Title/Abstract])) OR ("Multiple Sclerosis"[Mesh])) AND (("Anxiety"[Mesh]) OR ((((((((Anxiety[Title/Abstract]) OR (Angst[Title/Abstract])) OR (Nervousness[Title/Abstract])) OR (Hypervigilance[Title/Abstract])) OR (Anxiousness[Title/Abstract])) OR (Social Anxiety[Title/Abstract])) OR (Anxieties, Social[Title/Abstract])) OR (Social Anxieties[Title/Abstract])))) AND ((("Prevalence"[Mesh]) OR ((((((((Prevalence[Title/Abstract]) OR (Prevalences[Title/Abstract])) OR (Period Prevalence[Title/Abstract])) OR (Period Prevalences[Title/Abstract])) OR (Prevalence, Period[Title/Abstract])) OR (Point Prevalence[Title/Abstract])) OR (Point Prevalences[Title/Abstract])) OR (Prevalence, Point[Title/Abstract]))) OR (("Risk Factors"[Mesh]) OR (((((((Risk Factors[Title/Abstract]) OR (Factor, Risk[Title/Abstract])) OR (Risk Factor[Title/Abstract])) OR (Health Correlates[Title/Abstract])) OR (Correlates, Health[Title/Abstract])) OR (factor[Title/Abstract])) OR (factors[Title/Abstract])))) ("Multiple Sclerosis"[Title/Abstract] OR "sclerosis multiple"[Title/Abstract] OR "sclerosis disseminated"[Title/Abstract] OR "disseminated sclerosis"[Title/Abstract] OR (("ms"[Journal] OR "med sci paris"[Journal] OR "ms"[All Fields]) AND ("Multiple Sclerosis"[MeSH Terms] OR ("Multiple"[All Fields] AND "Sclerosis"[All Fields]) OR "Multiple Sclerosis"[All Fields])) OR "multiple sclerosis acute fulminating"[Title/Abstract] OR "Multiple Sclerosis"[MeSH Terms]) AND ("Anxiety"[MeSH Terms] OR ("Anxiety"[Title/Abstract] OR "Angst"[Title/Abstract] OR "Nervousness"[Title/Abstract] OR "Hypervigilance"[Title/Abstract] OR "Anxiousness"[Title/Abstract] OR "social anxiety"[Title/Abstract] OR (("Anxiety"[MeSH Terms] OR "Anxiety"[All Fields] OR "Anxieties"[All Fields] OR "anxiety s"[All Fields]) AND "Social"[Title/Abstract]) OR "social anxieties"[Title/Abstract])) AND ("Prevalence"[MeSH Terms] OR ("Prevalence"[Title/Abstract] OR "Prevalences"[Title/Abstract] OR "period

prevalence"[Title/Abstract] OR "period prevalences"[Title/Abstract] OR "prevalence period"[Title/Abstract] OR "point prevalence"[Title/Abstract] OR "point prevalences"[Title/Abstract] OR "prevalence point"[Title/Abstract]) OR ("Risk Factors"[MeSH Terms] OR ("Risk Factors"[Title/Abstract] OR "factor risk"[Title/Abstract] OR "risk factor"[Title/Abstract] OR "health correlates"[Title/Abstract] OR "correlates health"[Title/Abstract] OR "Factor"[Title/Abstract] OR "Factors"[Title/Abstract]))) 480 2:58:36

Embase

No. Query Results Date
#1 'multiple sclerosis'/exp 139804 16-May-21
#2 'multiple sclerosis':ab,ti OR 'sclerosis, multiple':ab,ti OR 'sclerosis, disseminated':ab,ti OR 'disseminated sclerosis':ab,ti OR (ms:ab,ti AND 'multiple sclerosis':ab,ti) OR 'multiple sclerosis, acute fulminating':ab,ti 124188 16-May-21
#3 'anxiety'/exp 234320 16-May-21
#4 anxiety:ab,ti OR angst:ab,ti OR nervousness:ab,ti OR hypervigilance:ab,ti OR anxiousness:ab,ti OR 'social anxiety':ab,ti OR 'anxieties, social':ab,ti OR 'anxiety, social':ab,ti OR 'social anxieties':ab,ti 301034 16-May-21
#5 'prevalence'/exp 810069 16-May-21
#6 prevalence:ab,ti OR prevalences:ab,ti OR 'period prevalence':ab,ti OR 'period prevalences':ab,ti OR 'prevalence, period':ab,ti OR 'point prevalence':ab,ti OR 'point prevalences':ab,ti OR 'prevalence, point':ab,ti 955535 16-May-21
#7 'risk factor'/exp 1113628 16-May-21
#8 'risk factors':ab,ti OR 'factor, risk':ab,ti OR 'risk factor':ab,ti OR 'health correlates':ab,ti OR 'correlates, health':ab,ti OR factor:ab,ti OR factors:ab,ti 4595379 16-May-21
#9 #1 OR #2 151790 16-May-21
#10 #3 OR #4 364569 16-May-21
#11 #5 OR #6 OR #7 OR #8 5788242 16-May-21
#12 #9 AND #10 AND #11 1102 16-May-21

Cochrane Library

ID Search
#1 MeSH descriptor: [Multiple Sclerosis] explode all trees
#2 (Multiple Sclerosis, Acute Fulminating):ti,ab,kw
#3 #1 #2
#4 MeSH descriptor: [Anxiety] explode all trees
#5 (Anxiety):ti,ab,kw OR (Angst):ti,ab,kw
#6 #4 or #5
#7 MeSH descriptor: [Prevalence] explode all trees
#8 MeSH descriptor: [Risk Factors] explode all trees
#9 #7 or #8
#10 #3 and #6 and #9

#11 MeSH descriptor: [Multiple Sclerosis] explode all trees
#12 MeSH descriptor: [Multiple Sclerosis] explode all trees
#13 MeSH descriptor: [Multiple Sclerosis] explode all trees
#14 MeSH descriptor: [Multiple Sclerosis] explode all trees
#15 (Multiple Sclerosis):ti,ab,kw OR (Sclerosis, Multiple):ti,ab,kw OR (Sclerosis, Disseminated):ti,ab,kw OR (Disseminated Sclerosis):ti,ab,kw OR (MS (Multiple Sclerosis)):ti,ab,kw
#16 (Multiple Sclerosis, Acute Fulminating):ti,ab,kw
#17 MeSH descriptor: [Anxiety] explode all trees
#18 (Anxiety):ti,ab,kw OR (Angst):ti,ab,kw OR (Nervousness):ti,ab,kw OR (Hypervigilance):ti,ab,kw OR (Anxiousness):ti,ab,kw
#19 (Social Anxiety):ti,ab,kw OR (Anxieties, Social):ti,ab,kw OR (Anxiety, Social):ti,ab,kw OR (Social Anxieties):ti,ab,kw
#20 MeSH descriptor: [Prevalence] explode all trees
#21 (Prevalence):ti,ab,kw OR (Prevalences):ti,ab,kw OR (Period Prevalence):ti,ab,kw OR (Period Prevalences):ti,ab,kw OR (Prevalence, Period):ti,ab,kw
#22 (Point Prevalence):ti,ab,kw OR (Point Prevalences):ti,ab,kw OR (Prevalence, Point):ti,ab,kw
#23 MeSH descriptor: [Risk Factors] explode all trees
#24 (Risk Factors):ti,ab,kw OR (Factor, Risk):ti,ab,kw OR (Risk Factor):ti,ab,kw OR (Health Correlates):ti,ab,kw OR (Correlates, Health):ti,ab,kw
#25 (factor):ti,ab,kw OR (factors):ti,ab,kw
#26 #14 OR #15 OR #16
#27 #17 OR #18 OR #19
#28 #20 OR #21 OR #22 OR #23 OR #24 OR #25
#29 #26 AND #27 AND #28

Web of Science

ID Results "Query Sort" By Filters

1 308,475

TOPIC: (Anxiety) OR TOPIC: (Angst) OR TOPIC: (Nervousness) OR TOPIC: (Hypervigilance) OR TOPIC: (Anxiousness) OR TOPIC: (Social Anxiety) OR TOPIC: (Anxieties, Social) OR TOPIC: (Anxiety, Social) OR TOPIC: (Social Anxieties) Indexes=SCI-EXPANDED, SSCI, A&HCI, CPCI-S, CPCI-SSH, BKCI-S, BKCI-SSH, ESCI, CCR-EXPANDED, IC Timespan=All years

2 135015 TOPIC: (Multiple Sclerosis) OR TOPIC: (Sclerosis, Multiple) OR TOPIC: (Sclerosis, Disseminated) OR TOPIC: (Disseminated Sclerosis) OR TOPIC: (MS (Multiple Sclerosis)) OR TOPIC: (Multiple Sclerosis, Acute Fulminating) Indexes=SCI-EXPANDED, SSCI, A&HCI, CPCI-S, CPCI-SSH, BKCI-S, BKCI-SSH, ESCI, CCR-EXPANDED, IC Timespan=All years

3 6540134 TOPIC: (Prevalence) OR TOPIC: (Prevalences) OR TOPIC: (Period Prevalence) OR TOPIC: (Period Prevalences) OR TOPIC: (Prevalence, Period) OR TOPIC: (Point Prevalence) OR TOPIC: (Point

Prevalences) OR TOPIC: (Prevalence, Point) OR TOPIC: (Risk Factors) OR TOPIC: (Factor, Risk) OR TOPIC: (Risk Factor) OR TOPIC: (Health Correlates) OR TOPIC: (Correlates, Health) OR TOPIC: (factor) OR TOPIC: (factors) Indexes=SCI-EXPANDED, SSCI, A&HCI, CPCI-S, CPCI-SSH, BKCI-S, BKCI-SSH, ESCI, CCR-EXPANDED, IC Timespan=All years

4 841 #1 AND #2 AND #3 Indexes=SCI-EXPANDED, SSCI, A&HCI, CPCI-S, CPCI-SSH, BKCI-S, BKCI-SSH, ESCI, CCR-EXPANDED, IC Timespan=All years
