# Supplementary material for: The prevalence and risk factors of anxiety in multiple sclerosis: A systematic review and meta-analysis
Source: Front Neurosci. 2023 Apr 17;17:1120541. doi: 10.3389/fnins.2023.1120541 (PMC10149809; doi:10.3389/fnins.2023.1120541)

Appendix 2. Supplementary figures

Figure S1. Forest plot of the effect of age at survey on anxiety in MS patients

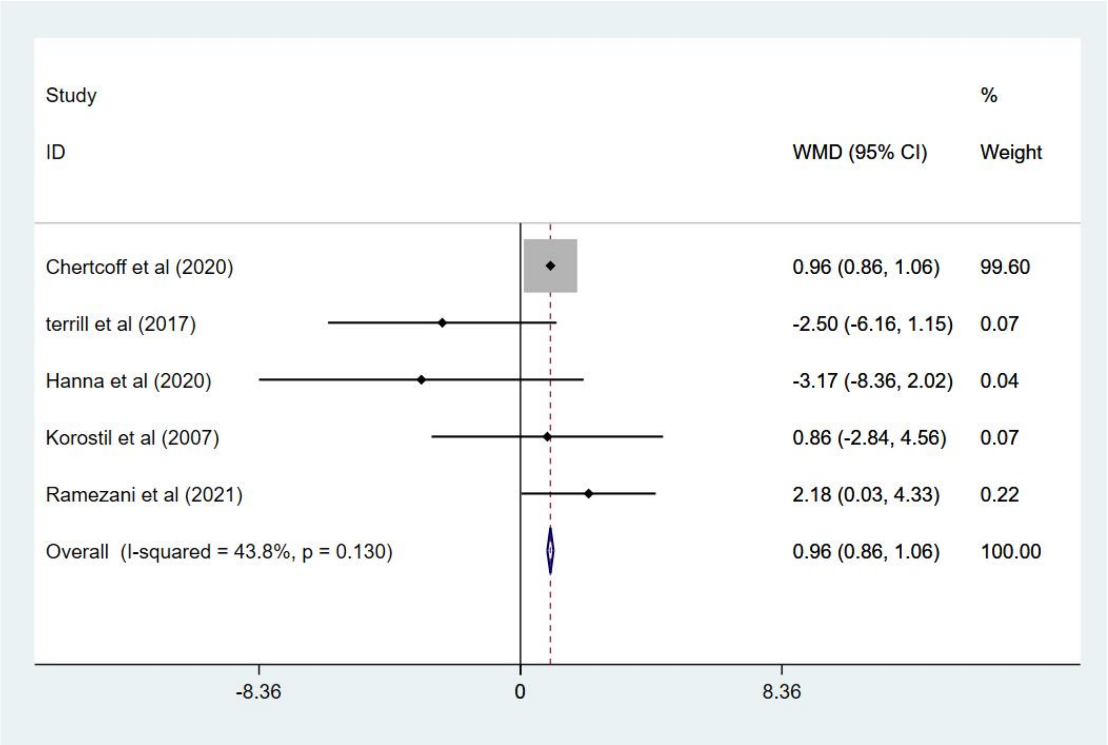

Figure S2. Forest plot of the effect of gender (female) on anxiety in MS patients

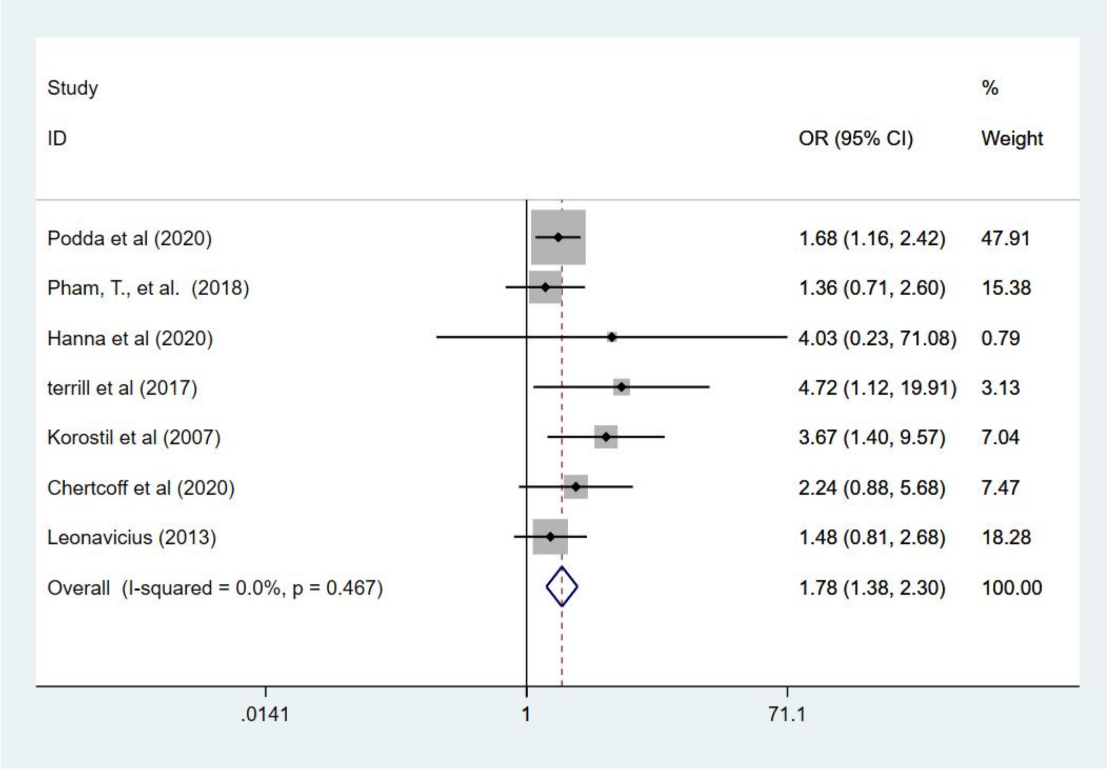

Figure S3. Forest plot of the effect of depression on anxiety in MS patients

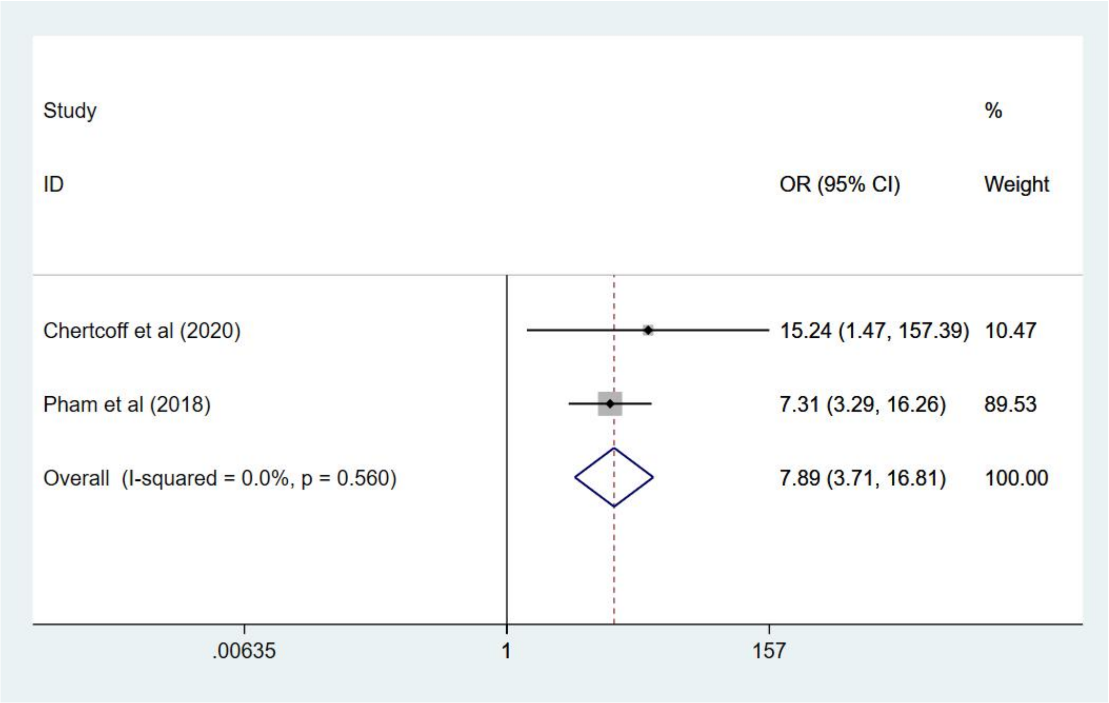

Figure S4. Forest plot of the effect of marriage on anxiety in MS patients

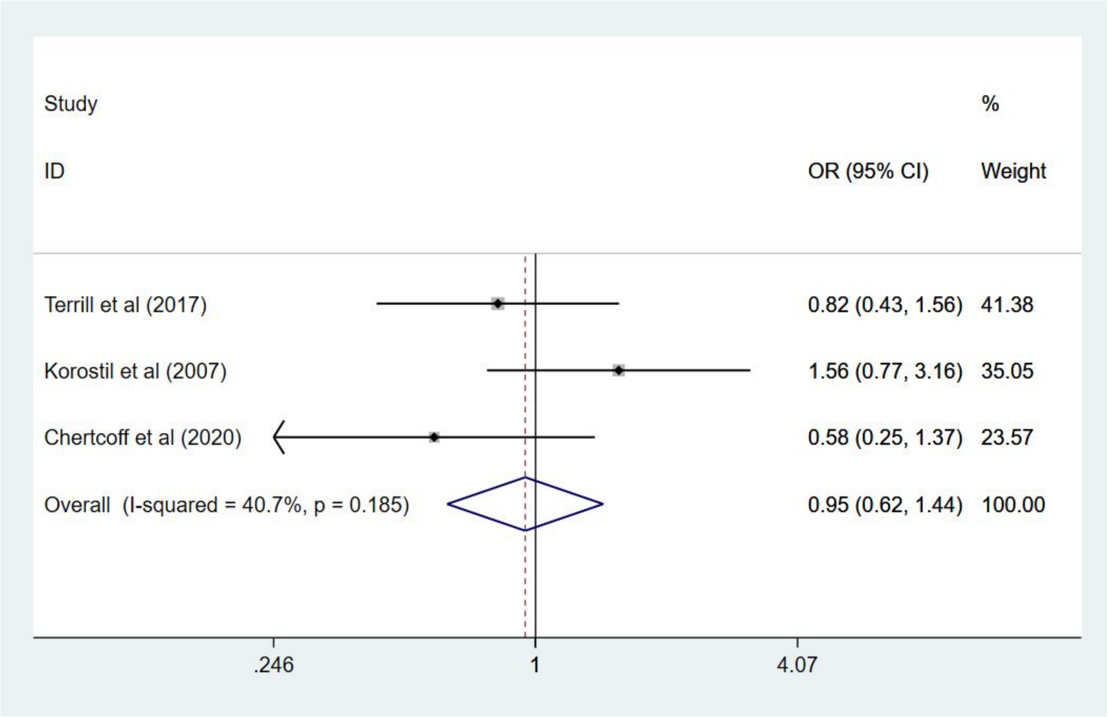

Figure S5. Forest plot of the effect of employment status on anxiety in MS patients

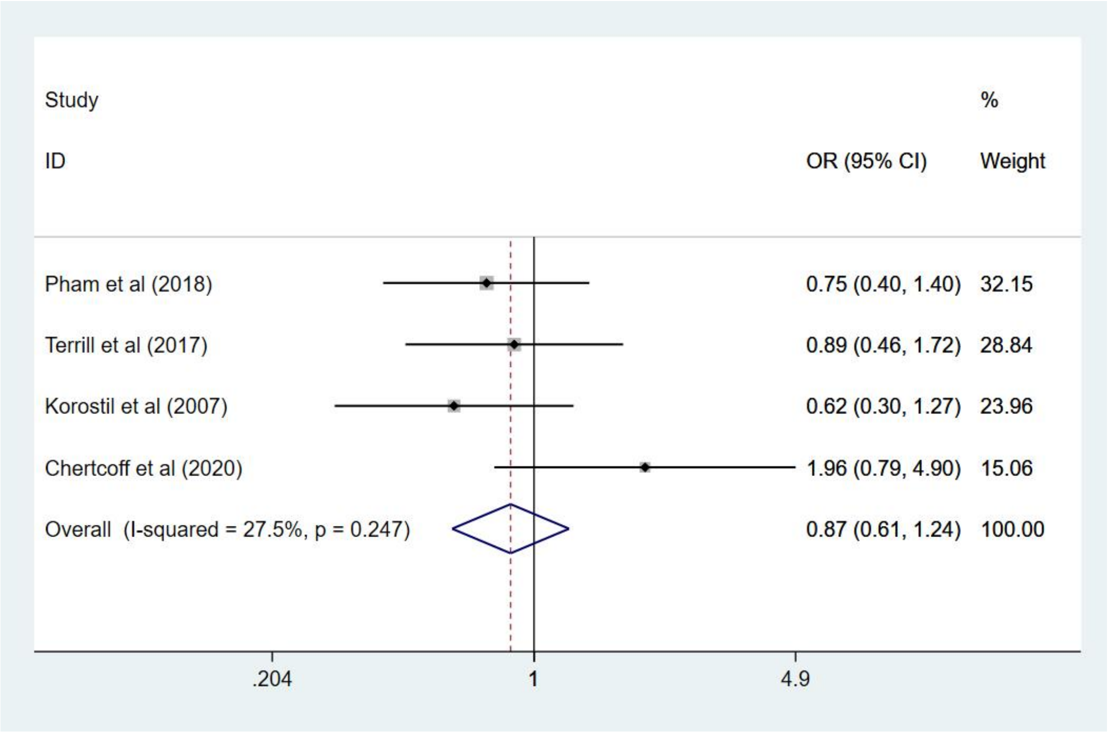

Figure S6. Forest plot of the effect of years of education on anxiety in MS patients

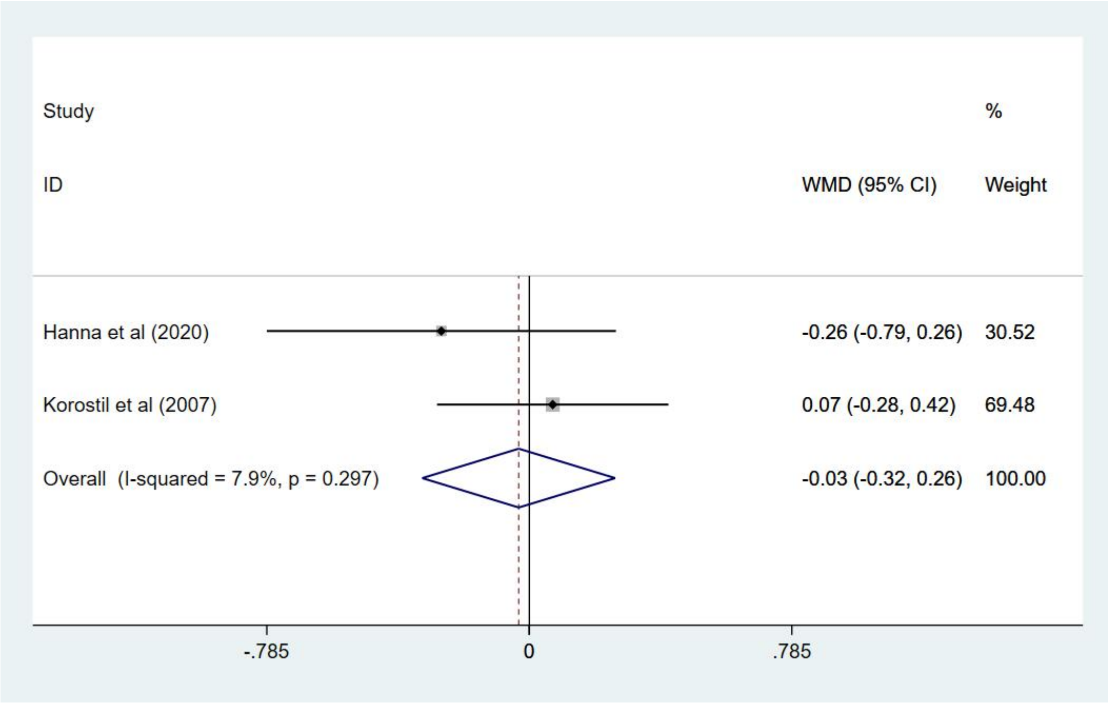

Figure S7. Forest plot of the effect of education levels on anxiety in MS patients

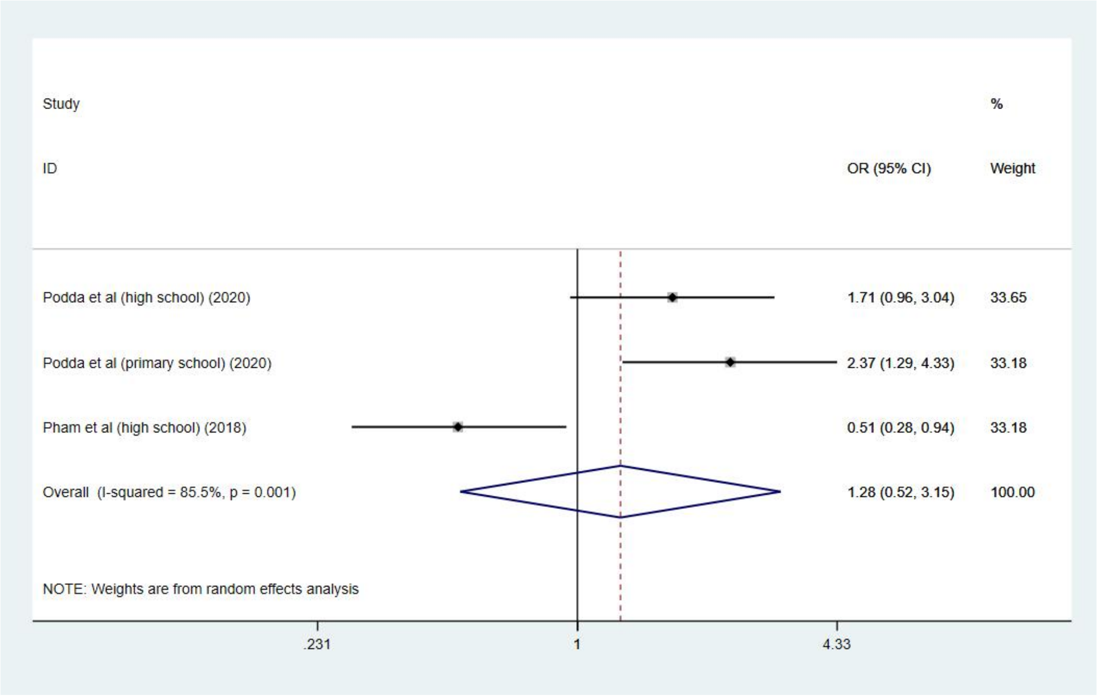

Figure S8. Forest plot of the effect of disease duration on anxiety in MS patients

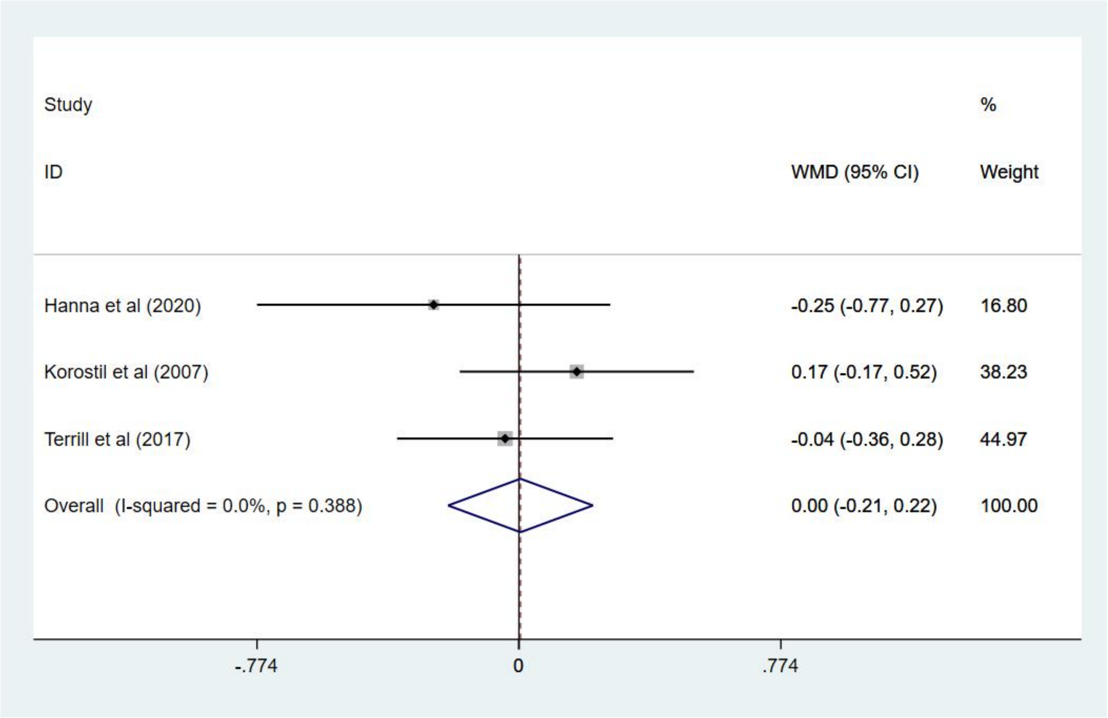

Figure S9. Forest plot of the effect of disease course (relapsing remitting MS) on anxiety in MS patients

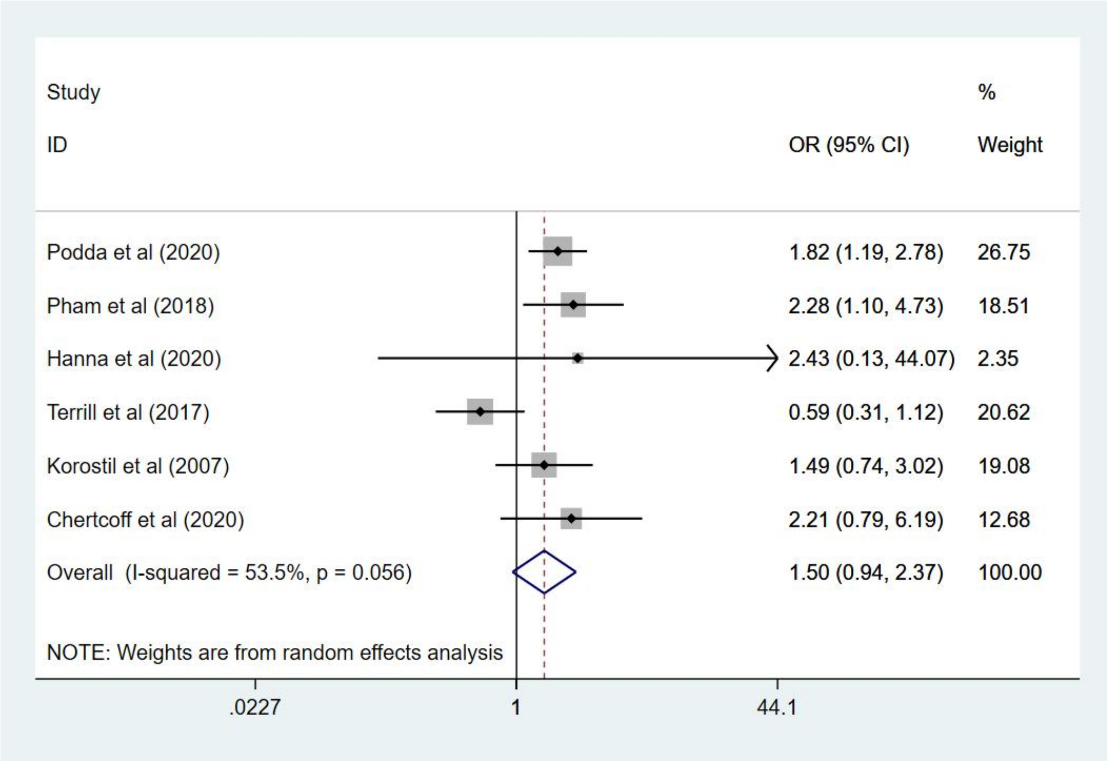

Figure S10. Forest plot of the effect of past psychiatric history on anxiety in MS patients

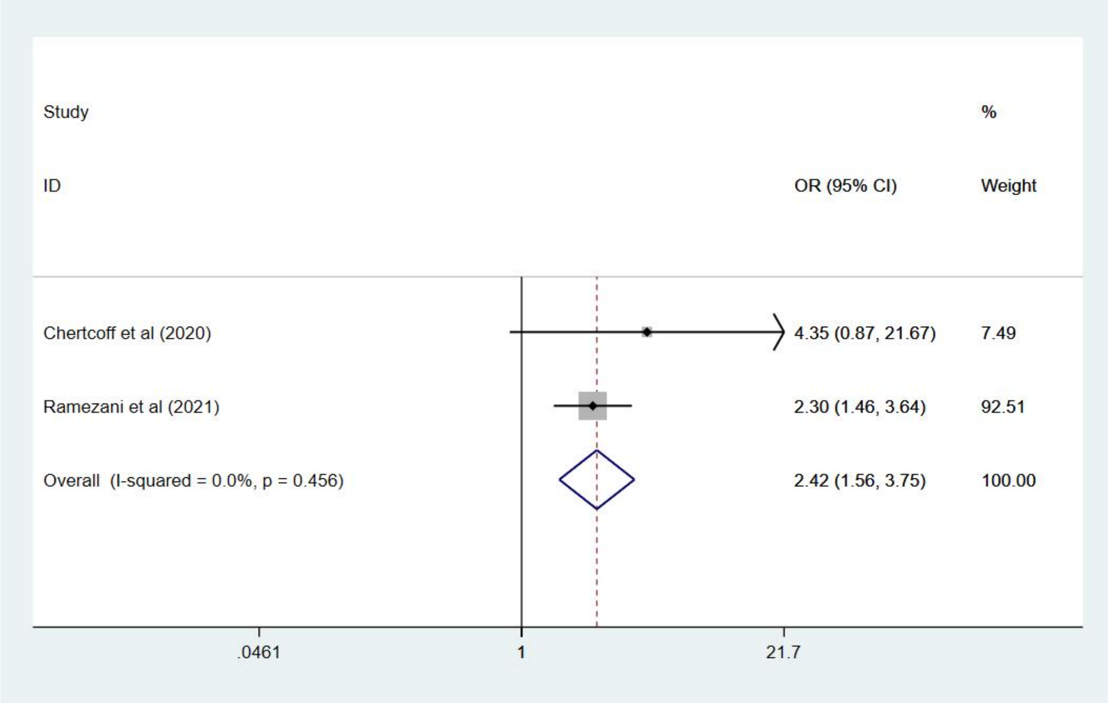

Figure S11. Forest plot of the effect of living together on anxiety in MS patients

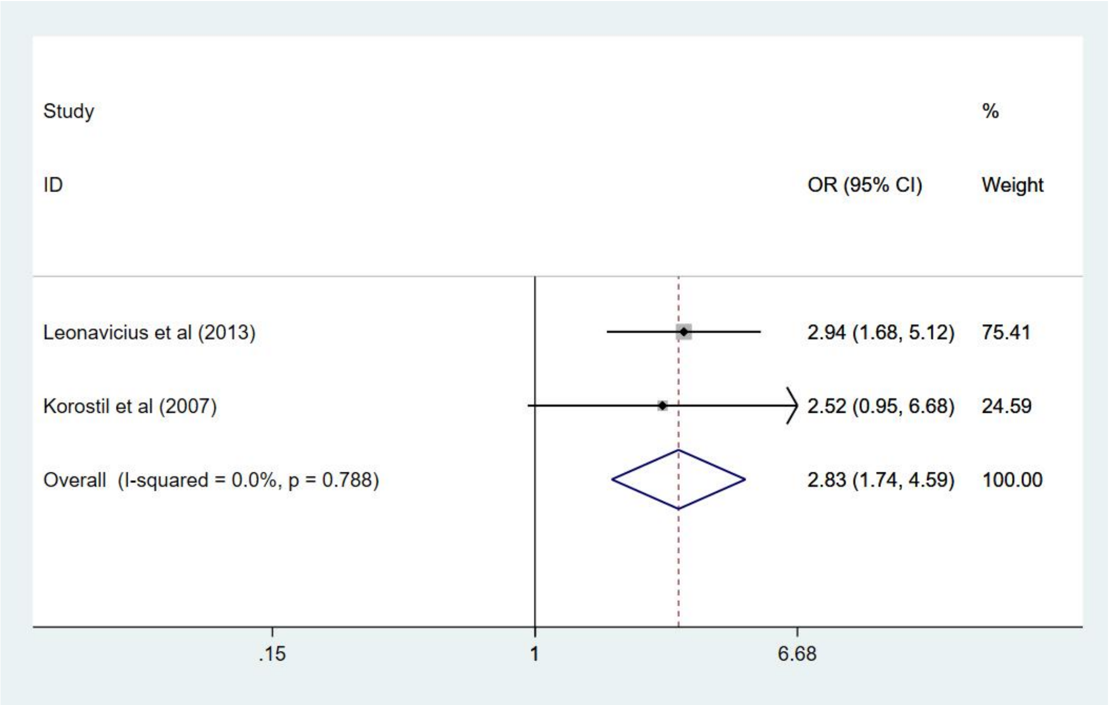

Figure S12. Forest plot of the effect of family psychiatric history on anxiety in MS patients

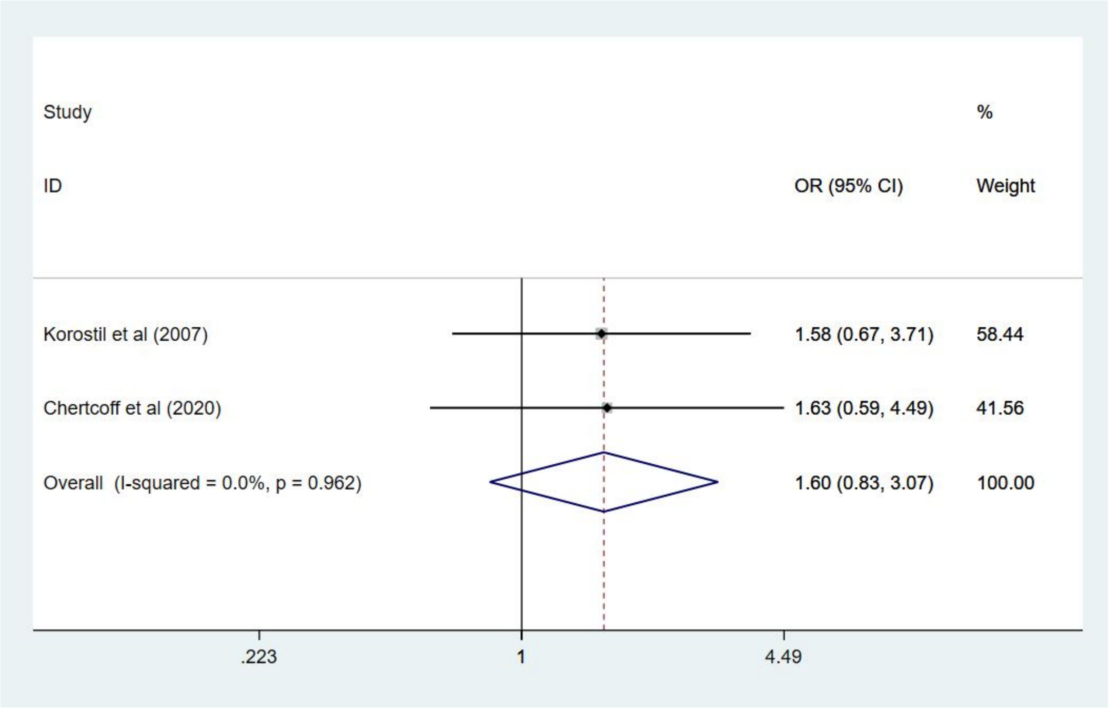

Figure S13. Forest plot of the effect of baseline EDSS on anxiety in MS patients

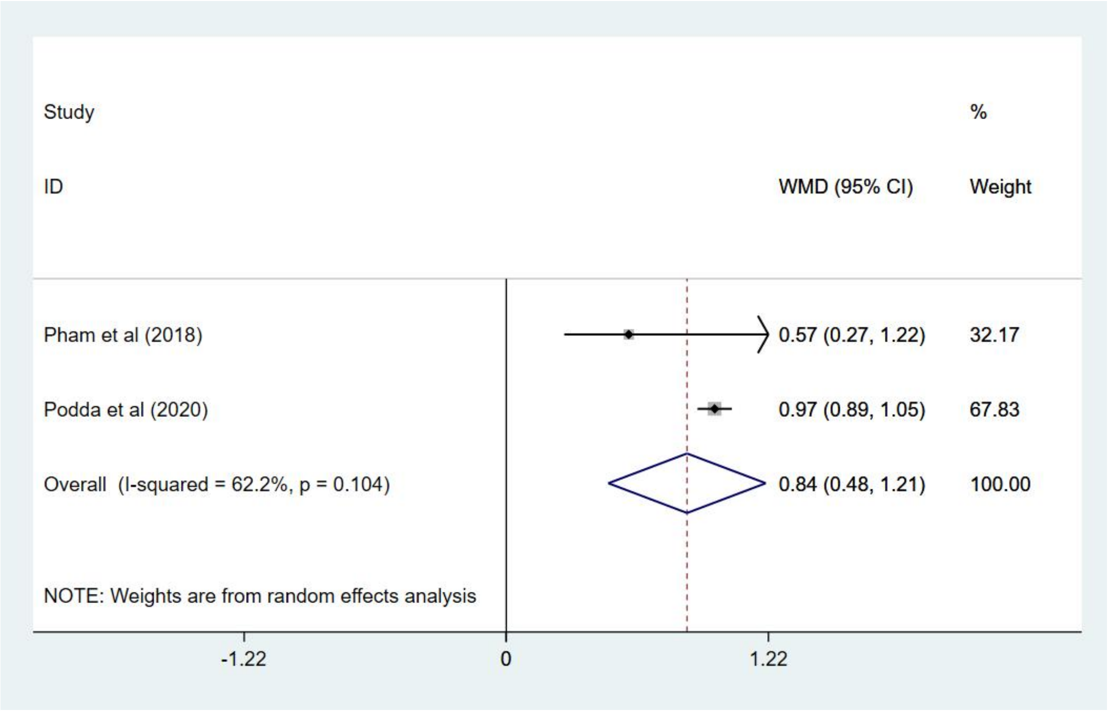

Figure S14. Forest plot of the effect of Disease modifying therapy on anxiety in MS patients

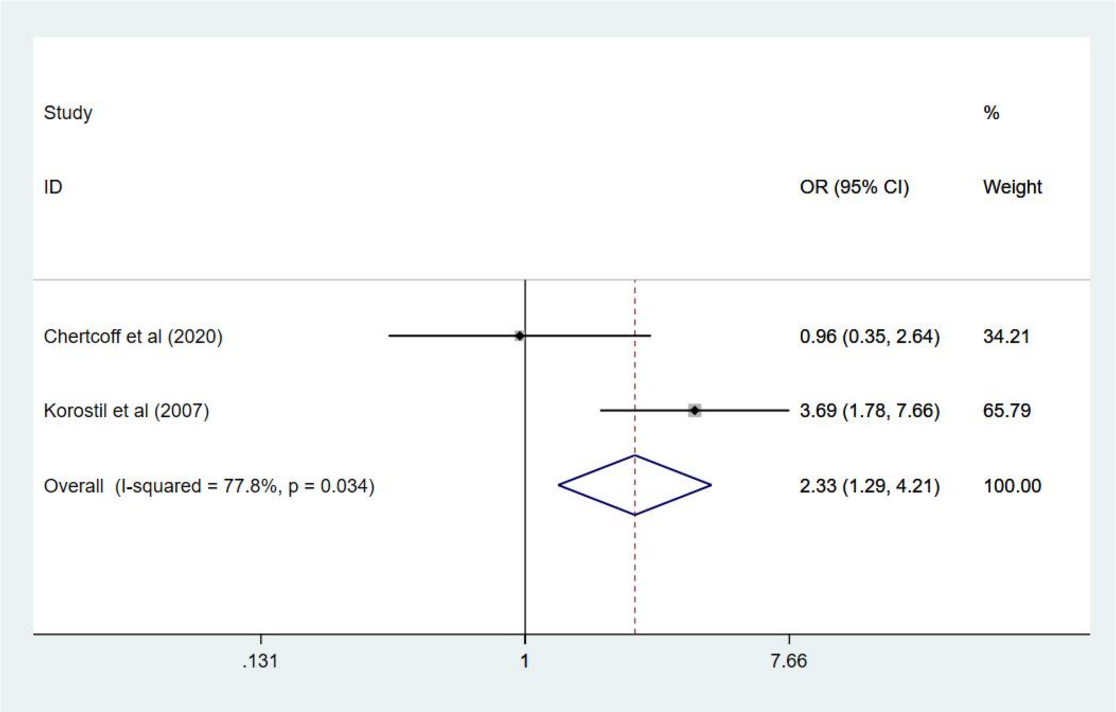

Supplement: Supplementary file 2 [file Data_Sheet_2.pdf]
